# Fungal Melanins as Potential Reactive Oxygen Species-Scavenging Neuroprotective Agents

**DOI:** 10.3390/molecules31173135

**Published:** 2026-09-07

**Authors:** Vy D. A. Nguyen, Yen T. H. Tran, Debby Mangelings, Yvan Vander Heyden, Ann Van Eeckhaut, Hanh T. M. Tran

**Affiliations:** 1 Applied Microbiology Laboratory, School of Biotechnology, International University-VNU HCM, Linh Xuan Ward, Ho Chi Minh City 71309, Vietnam; vynda24@mp.hcmiu.edu.vn (V.D.A.N.); tthyen@hcmiu.edu.vn (Y.T.H.T.); 2 Research Group Analytical Chemistry, Applied Chemometrics and Molecular Modelling, Vrije Universiteit Brussel (VUB), Laarbeeklaan 103, 1090 Brussels, Belgium; debby.mangelings@vub.be (D.M.); yvan.vander.heyden@vub.be (Y.V.H.); 3 Research Group Experimental Pharmacology (EFAR), Department of Pharmaceutical Chemistry, Drug Analysis and Drug Information (FASC), Center for Neurosciences (C4N), Vrije Universiteit Brussel (VUB), Laarbeeklaan 103, 1090 Brussels, Belgium; 4 Research Group BIONUC (Biotechnology of Nutraceuticals and Bioactive Compounds), Department of Functional Biology, Microbiology Area, IUOPA and ISPA, Faculty of Medicine, University of Oviedo, 33006 Oviedo, Spain

**Keywords:** melanin-producing fungi, neuroprotection, hydrogen peroxide (H_2_O_2_), 1-methyl-4-phenylpyridinium (MPP^+^), SH-SY5Y cells

## Abstract

Oxidative stress is strongly associated with neuronal damage in neurodegenerative diseases, such as Parkinson’s disease (PD). Fungal melanins are remarkable free radical scavengers; however, their capacity to protect neurons from reactive oxygen species (ROS)-induced damage remains understudied. This research evaluated the neuroprotective effects of fungal melanins and their arginine-modified counterparts on SH-SY5Y cells against neurotoxins, as well as their impact on ROS levels. Exposure to 0.6 mM H_2_O_2_ or 1 mM MPP^+^ markedly elevated ROS levels and reduced cell viability to approximately 56% and 60%, respectively. At 10 μg/mL, melanins from *Apioperdon pyriforme*, *Russula nigricans*, and *Xylaria nigripes* significantly protected cells against H_2_O_2_ cytotoxicity, whereas arginine-modified melanin from the skin of *Scleroderma sinnamariense* significantly attenuated MPP^+^ cytotoxicity. Further dose-dependent evaluation revealed that melanin from *A. pyriforme* (10–12 μg/mL) displayed activity comparable to the positive control (164 μg/mL N-Acetylcysteine (NAC)) against H_2_O_2_ by increasing cell viability up to 90%. Similarly, arginine-modified melanin from *S. sinnamariense* (6–10 μg/mL) and NAC showed a comparable protective effect against MPP^+^, boosting cell viability up to 80%. Both samples suppressed ROS to levels comparable to, or lower than, the untreated control. Melanin from *A. pyriforme* is a promising candidate for further research into complementary therapies for PD.

## 1. Introduction

Neurodegenerative disorders (NDs) are one of the world’s major health concerns [1]. Among these disorders, Parkinson’s disease (PD) is one of the most prevalent and rapidly growing [1]. Worldwide, the number of PD cases has increased nearly fourfold, from 3.15 million in 1990 to 11.77 million in 2021 [2], and is anticipated to reach around 25.2 million by 2050 [3]. The increase in the incidence of NDs has become an alarming issue, posing a global health burden on both individuals and society.

Oxidative stress (OS) has been identified as one of the central mechanisms related to the pathogenesis and progression of NDs, such as PD [4]. An imbalance between endogenous antioxidant species and the level of reactive oxygen species (ROS) (such as hydrogen peroxide ($H_{2}O_{2}$), superoxide ($O_{2}^{∙-}$), and hydroxyl (${\mathrm{OH}}^{∙}$) radicals) leads to the unregulated production of ROS, eventually resulting in OS [5]. Accounting for roughly 20% of the body’s oxygen consumption, while possessing limited endogenous defenses, the brain is exceptionally vulnerable to OS [6]. OS may damage not only biomolecules (e.g., DNA, RNA, lipids, and protein) but also cell structures (e.g., mitochondrial membranes and electron transport chain complexes), further increasing mitochondrial ROS production and establishing a vicious cycle of oxidative damage and mitochondrial dysfunction [5,6]. Consequently, this persistent impairment causes bioenergetic depletion, mitochondrial dysfunction, and oxidative damage, eventually contributing to the progression of NDs, particularly the degeneration of neurons in PD [7].

Currently, there are no cures for NDs, including PD. The majority of the existing treatments focus on symptom relief, though some aim at disease prevention or slowing progression [8]. Given the central role of OS in NDs, particularly PD, where oxidative injury drives the selective loss of dopaminergic neurons, dietary antioxidants present a promising strategy for neuroprotection [4,9]. These compounds directly neutralize ROS and induce endogenous protective proteins, thereby maintaining cellular homeostasis [9]. Among the endogenous antioxidants, melanins have been known for their remarkable antioxidant, potent free-radical-scavenging, and metal-chelating properties [10]. Melanins are highly heterogeneous polymers found in members of all biological kingdoms and serve critical roles in photoprotection, antioxidant defense, and metal sequestration [10]. Based on their biosynthetic precursors, melanins can be broadly classified into eumelanin, pheomelanin, allomelanin, and pyomelanin. Eumelanin is primarily derived from 3,4-dihydroxy-L-phenylalanine (L-DOPA) and consists mainly of 5,6-dihydroxyindole (DHI) and 5,6-dihydroxyindole-2-carboxylic acid (DHICA) units; pheomelanin contains sulfur and is typically formed from benzothiazine and benzothiazole monomers; allomelanin comprises nitrogen-free pigments derived mainly from catechol or 1,8-dihydroxynaphthalene (1,8-DHN); and pyomelanin is produced via homogentisic acid accumulation during tyrosine degradation [10]. Generally, the presence of persistent unpaired electrons gives melanins their characteristic paramagnetic properties and contributes to their powerful redox and antioxidant activities [10,11].

In humans, melanin is present in several forms, such as eumelanin and pheomelanin, which are found primarily in the skin, hair, and eyes, and neuromelanin (NM), a specialized pigment that accumulates mostly in the substantia nigra and locus coeruleus of the brain [12]. NM serves vital functions in the brain including assisting neurotransmission, regulating OS, modulating protein aggregation, and mediating metal sequestration and chelation [11,13]. Due to the paramagnetic nature of melanin, NM exerts neuroprotective effects by scavenging free radicals, chelating metal ions and toxins, sequestering them into stable complexes, and thereby limiting neuronal toxicity [14]. Under physiological conditions, NM acts as a protective neuro-shield by chelating excess iron and other redox-active metal ions, thereby limiting ROS generation and mitigating oxidative damage [15]. However, under pathological conditions, factors such as excessive iron accumulation, age-related alterations in the redox equilibrium of its DHI and DHICA subunits, and progressive degeneration of NM-containing dopaminergic neurons can compromise the metal-binding capacity and redox stability of NM [11]. NM is considered neuroprotective under normal conditions, whereas its detrimental effects arise only after neuronal homeostasis has been disrupted, thereby contributing to the progression rather than the initiation of neurodegeneration [15]. Consistent with this, the progressive loss of NM-containing dopaminergic neurons in the substantia nigra is a pathological hallmark of PD, indicating that depletion of functional NM, rather than its physiological presence, is associated with disease progression [16]. The potential loss of NM-mediated neuroprotection under pathological conditions has prompted interest in dietary melanins as complementary agents that may help compensate for impaired antioxidant defenses [17,18] and possibly reduce OS associated with neurodegeneration.

Berliner et al. (1993) first proposed the therapeutic use of microbial recombinant melanin and melanin-related compounds for NDs, suggesting that increasing melanin levels in nervous tissue could protect against toxin-induced neuronal damage and promote neuronal recovery [19]. This hypothesis has since been reinforced by evidence that the loss or dysfunction of endogenous NM during neurodegeneration compromises its antioxidant and metal-buffering functions [16]. Accordingly, eumelanin-based formulations are now being explored as biomaterials to restore these protective functions, highlighting their therapeutic potential for targeting OS-associated diseases such as NDs [11].

Melanin can be produced through synthetic methods or extracted from natural sources [10]. Among the natural sources, melanin production using fungi and bacteria is increasingly favored because it enables rapid, scalable production with minimal land requirements and relatively simple cultivation processes [20,21]. Fungi offer advantages over bacteria for melanin production, primarily because of their higher biomass yields [21]. Several melanin-producing fungal species have also been explored as functional foods or natural food additives [18,22] including *Auricularia auricula* (wood ear), *Pleurotus* spp. (oyster mushroom), *Tuber melanosporum* (black truffle), and *Inonotus obliquus* (Chaga) [23,24,25,26]. In vitro evaluations have consistently revealed the high antioxidant potential of fungal melanins [21]. In addition, fungal melanins also possess the ability to effectively protect different types of cells from different toxins. For example, a treatment with *A. aricula* melanin (1.6 mg/mL) restored human L02 hepatocyte cell viability to 98% and improved cell morphology under induced OS by 0.2 mM H_2_O_2_ [23]. Our previous studies also indicated that melanins from mature fruiting bodies of *Calvatia craniiformis*, *Daedaleopsis tricolor*, *Fomes fomentarius*, and *Xylaria plebeja* significantly protected SH-SY5Y cell viability against 1 mM H_2_O_2_-induced toxicity, increasing cell viability by up to 20% at 100 μg/mL [27,28].

Melanins are mostly insoluble in water and common organic solvents [10,29], which limits their applications. Previous studies suggested that amino acid modification of melanins can enhance their water solubility and some of their biological activities such as antioxidant activity and neuroprotective effects [10,30]. For example, arginine modification of *Lachnum* YM-346 melanin significantly enhanced both its water solubility and the antioxidant capacity [31]. On the other hand, arginine-modified melanins from *D. tricolor*, *F. fomentarius*, and *X. plebeja* showed neuroprotective effects against 0.2 mM 1-methyl-4-phenyl-1,2,3,6-tetrahydropyridine (MPTP) toxicity, while their native forms exhibited no significant activity [27,28].

Melanin production in fungi is a dynamic and complex process largely influenced by the fungal developmental stages (mycelium vs. fruiting body), leading to pigments with distinct physicochemical and biological properties [32,33,34]. While most previous studies on fungal melanin have focused on mycelia, melanin derived from fungal fruiting bodies remains largely unexplored [35]. In addition, although the neuroprotective potential of certain fungal melanins against neurotoxins has been initially assessed, their protective mechanisms remain poorly understood. This study evaluates the comparative cytoprotective efficacy and ROS-scavenging capacity of native and arginine-modified fungal melanins against H_2_O_2_- and MPP^+^-induced cytotoxicity in SH-SY5Y cells. It should be noted that MPP^+^ is widely utilized to establish in vitro models of PD because it induces dopaminergic neuronal cell death. Therefore, the main objectives of this study were to: (1) collect and molecularly identify potent melanin-producing fungi from diverse locations, followed by melanin extraction, purification, and subsequent arginine modification to enhance water solubility; (2) investigate the neuroprotective effects of the fungal melanins and arginine-modified melanins on SH-SY5Y human neuroblastoma cells against neurotoxins (H_2_O_2_ and MPP^+^) via the MTT assay; and (3) initially assess the ROS-scavenging capacity of potent melanins using 2′,7′-dichlorodihydrofluorescein diacetate (DCFH-DA).

## 2. Results

### 2.1. List of Melanin-Producing Fungal Species

The melanin-producing fungi were selected based on the color of their mature fruiting bodies (brown to black), indicating the presence of melanin. In addition, they either produce large fruiting bodies or numerous relatively small fruiting bodies in the field (Supplementary Figure S1) [36]. The fungi were classified based on morphological and ITS sequence analysis [37]. The BLAST results against the GenBank database are depicted in Supplementary Tables S1 and S2.

Ten melanins from nine fungal species, with *Scleroderma sinnamariense* providing both skin- and spore-derived pigments, were extracted and purified as described in the Method Section. The structural identity of the purified melanin was assessed though UV–Vis and FTIR spectroscopy in our previous work, confirming all extracted samples from the selected fungal species were indeed melanins [38]. A list of the melanin-producing fungal species along with their melanin yield and color is presented in Table 1.

As shown in Table 1, melanin yields extracted from nine fungal species varied considerably. The highest yield of purified melanin was from *F. fomentarius* with 99.83 mg g^−1^ of dried fungal biomass, followed by *A. pyriforme* at 90.16 mg g^−1^ of dried fungal biomass. Other promising sources of melanins are *R. nigricans* and *C. gigantea*, with yields of melanins of 78.65 and 76.91 mg g^−1^ of dried fungal biomass, respectively. Lower yields of melanins were observed in *D. concentrica* and the spore *S. sinnamariense*, at approximately 33.10 and 34.39 mg g^−1^ of dried fungal biomass, respectively. In contrast, the skin of *S. sinnamariense* was roughly six times lower than that of the spore, at only 6.72 mg g^−1^ of dried fungal biomass. Other fungi species producing relatively low melanin yields are *A. asiaticus* and *D. tricolor,* reaching only 16.57 and 10.74 mg g^−1^ of dried fungal biomass. The lowest yield of melanins was extracted from *X. nigripes*, with only 3.46 mg g^−1^ of dried fungal biomass.

Melanins from previously studied fungal species also differed significantly from one species to another. Under optimal conditions, *Paecilomyces variotii* mycelium and dry powder of *A. auricula* mushroom waste generated yields of about 108 mg g^−1^ and 110 mg g^−1^, respectively, which are comparable with those of *F. fomentarius* and *A. pyriforme* in our research [23,39]. The melanin yields obtained from *D. concentrica* and the spore of *S. sinnamariense* in the current study were comparable to those reported for the yellow and red varieties of *Stropharia rugosoannulata* (35 and 40 mg g^−1^ purified melanin, respectively) [40].

To evaluate their neuroprotective potential against H_2_O_2_- and MPP^+^-induced oxidative stress in SH-SY5Y cells, melanins and arginine-modified melanins from *A. pyriforme*, *R. nigricans*, *S. sinnamariense* (skin and spore), and *X. nigripes* were selected for the initial screening. These melanins were chosen based on a combination of high antioxidant activity [38], favorable extraction yields (*A. pyriforme* and *R. nigricans*), and biomass availability (skin and spore of *S. sinnamariense* and *X. nigripes*). More importantly, all five selected candidates demonstrated compatible solubility in 0.25 M NaOH and ease of chemical modification with arginine as compared to other fungal melanin samples.

### 2.2. Effects of Different Concentrations of Melanins and Arginine-Modified Melanins on SH-SY5Y Cell Viability

**Figure 1:**
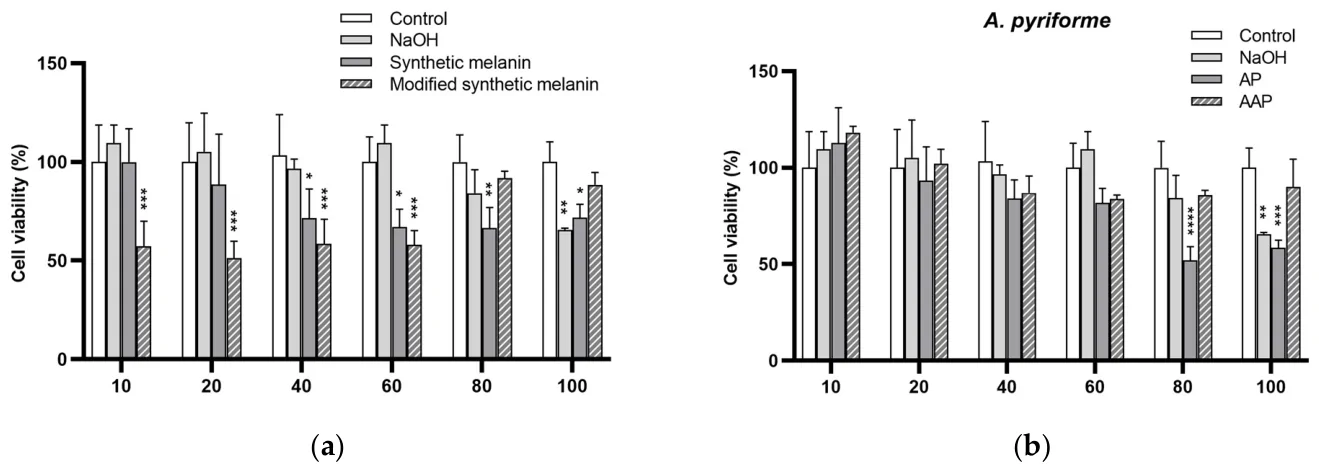

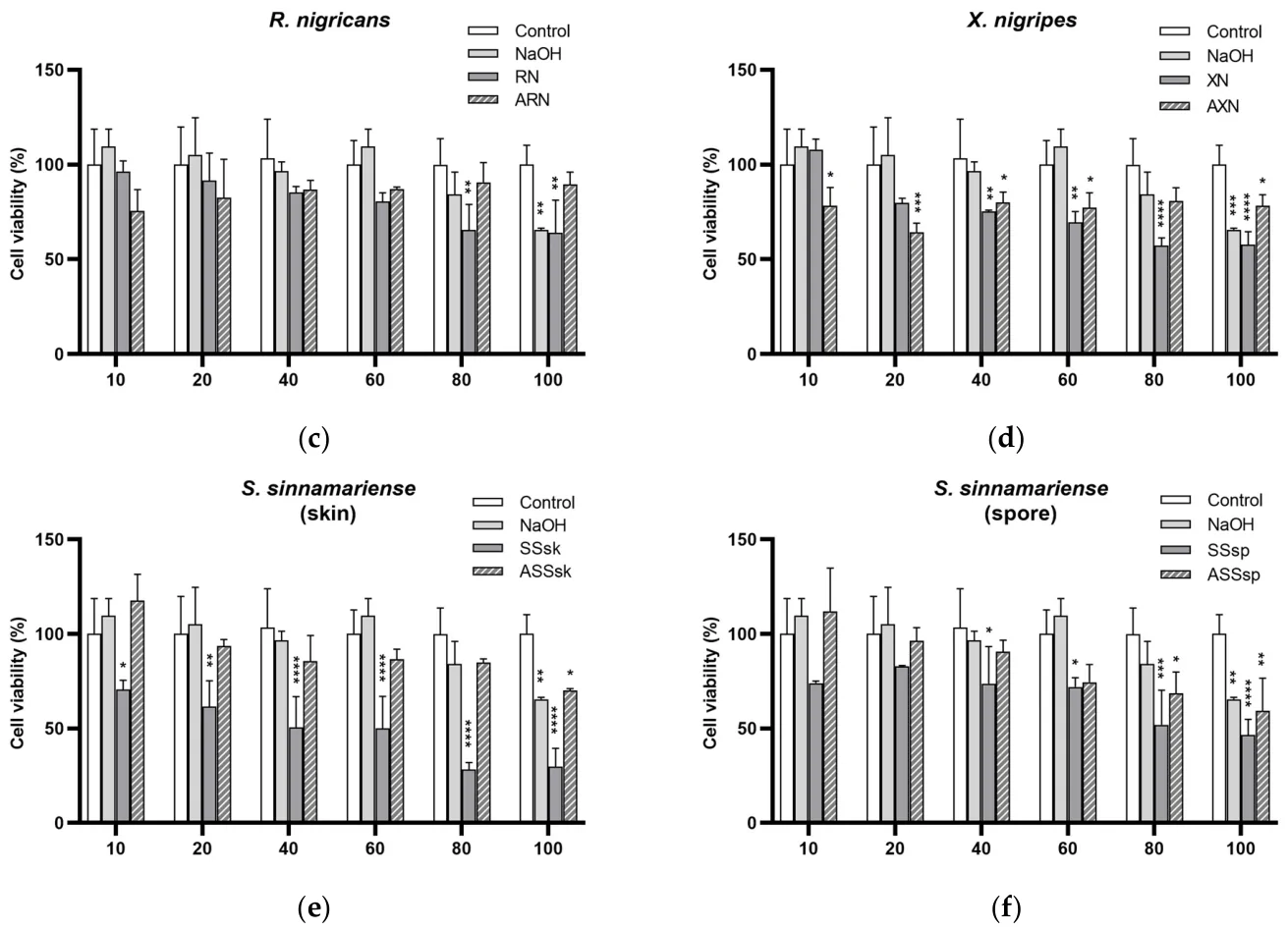
Effects of melanins and arginine-modified melanins from different fungal species on SH-SY5Y cell viability in 24 h: (**a**) synthetic/commercial melanin; (**b**) *A. pyriforme*; (**c**) *R. nigricans*; (**d**) *X. nigripes*; (**e**) *S. sinnamariense* (skin); and (**f**) *S. sinnamariense* (spore). Statistical comparison between the treatment at each concentration and the corresponding control group (untreated cells) is denoted as follows: * *p* < 0.05, ** *p* < 0.01, *** *p* < 0.001, and **** *p* < 0.0001, Dunnett’s test. Data shown are mean ± SD (n = 3 independent experiments performed in triplicate). **Vehicle control group** (**NaOH**): consisted of NaOH at the concentrations (250, 500, 1000, 1500, 2000, or 2500 μM) corresponding to the concentrations present in melanin solutions (10, 20, 40, 60, 80, 100 μg/mL) in the melanin treatments. **Control**: cells in normal medium without melanin, modified melanin, and NaOH. **STM** and **ASTM**: synthetic melanin and modified synthetic melanin; **RN** and **ARN**: melanin and modified melanin from *R. nigricans*; **XN** and **AXN**: melanin and modified melanin from *X. nigripes*; **AP** and **AAP**: melanin and modified melanin from *A. pyriforme*; **SSsk** and **ASSsk**: melanin and modified melanin from skin of *S. sinnamariense*; **SSsp** and **ASSsp**: melanin and modified melanin from spore of *S. sinnamariense*. **White bar**: untreated cells/cells without H_2_O_2_; **black bar**: cells treated with H_2_O_2_ without sample.

SH-SY5Y cells were incubated with different concentrations of melanins or arginine-modified melanins for 24 h, and their effects on SH-SY5Y cell viability were assessed by the MTT assay. The obtained data are shown in Figure 1.

Overall, both melanins and modified melanins exhibited dose- and species-dependent effects on SH-SY5Y cell viability, and increasing concentrations generally resulted in greater decreases in cell viability, although the extent of this response varied among the fungal species, and modified melanins generally seemed less toxic than their counterparts.

At low concentrations (10 and 20 μg/mL), most melanin samples showed no effect on the viability of SH-SY5Y cells. However, at 10 μg/mL, except for melanin of *S. sinnamariense* (skin), which significantly reduced cell viability to 70%, most treatments in this concentration were not statistically different from untreated cells and maintained viability above 70%, suggesting 10 μg/mL as the baseline concentration for further testing. Starting at 40 μg/mL, significant cytotoxic effects were recorded with melanin from skin and spore of *S. sinnamariense* and *X. nigripes*. At 80 μg/mL, all melanin samples reduced cell viability to less than 70%. At 100 µg/mL, marked toxicity was observed in all melanin samples, and in the vehicle control (which contained 2500 μM NaOH, equivalent to the concentration of NaOH present in 100 µg/mL melanin).

Regarding modified melanins, only synthetic melanin and arginine-modified melanin from *X. nigripes* significantly reduced cell viability at 10 μg/mL. Interestingly, while the toxicity effect of the latter is persistent at higher concentrations, those of the former reduced as its concentration increased (Figure 1). In contrast, arginine-modified melanins from other species only exhibited significant toxicity at 80 μg/mL or above. Most modified melanins remained near 80% viability at 80 μg/mL, except for the modified melanin from *S. sinnamariense* (spore), which reduced cell viability to 68%. Interestingly, the arginine-modified melanins from the skin and spore of *S. sinnamariense* showed no significant toxicity within the 10–60 μg/mL range, contrasting sharply with their unmodified forms, which reduced cell viability across these concentrations. Moreover, none of the tested concentrations of arginine-modified melanins from *A. pyriforme* and *R. nigricans* showed significant effects on cell viability.

As previously noted, no significant reduction in cell viability was observed at 10 μg/mL for most samples. Therefore, this concentration was selected for the neuroprotective effect evaluation of the samples.

### 2.3. Screening for Potent Melanins and Arginine-Modified Melanins with Cell-Protective Effects on SH-SY5Y Cells Against H_2_O_2_ Cytotoxicity

**Figure 2:**
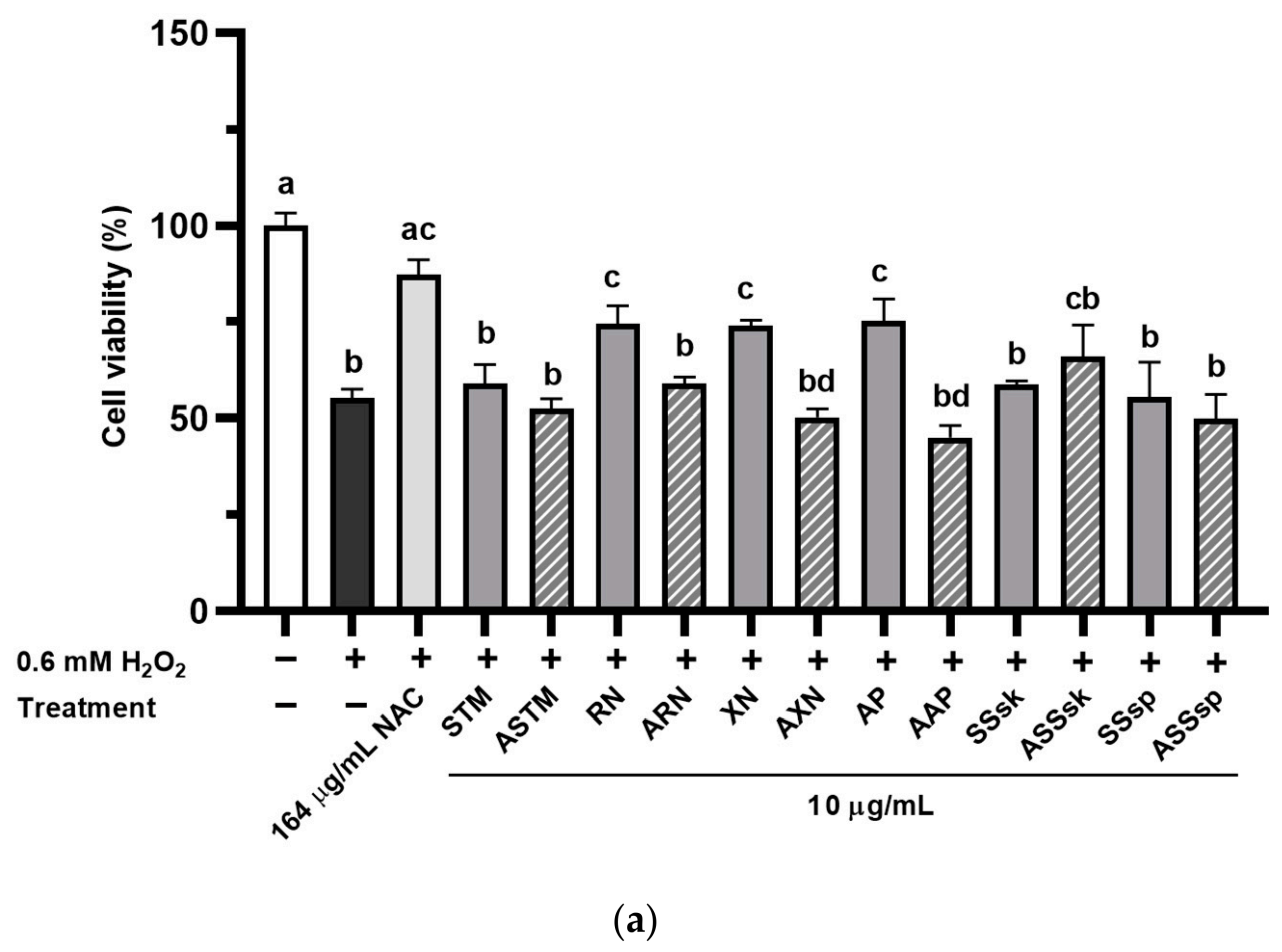

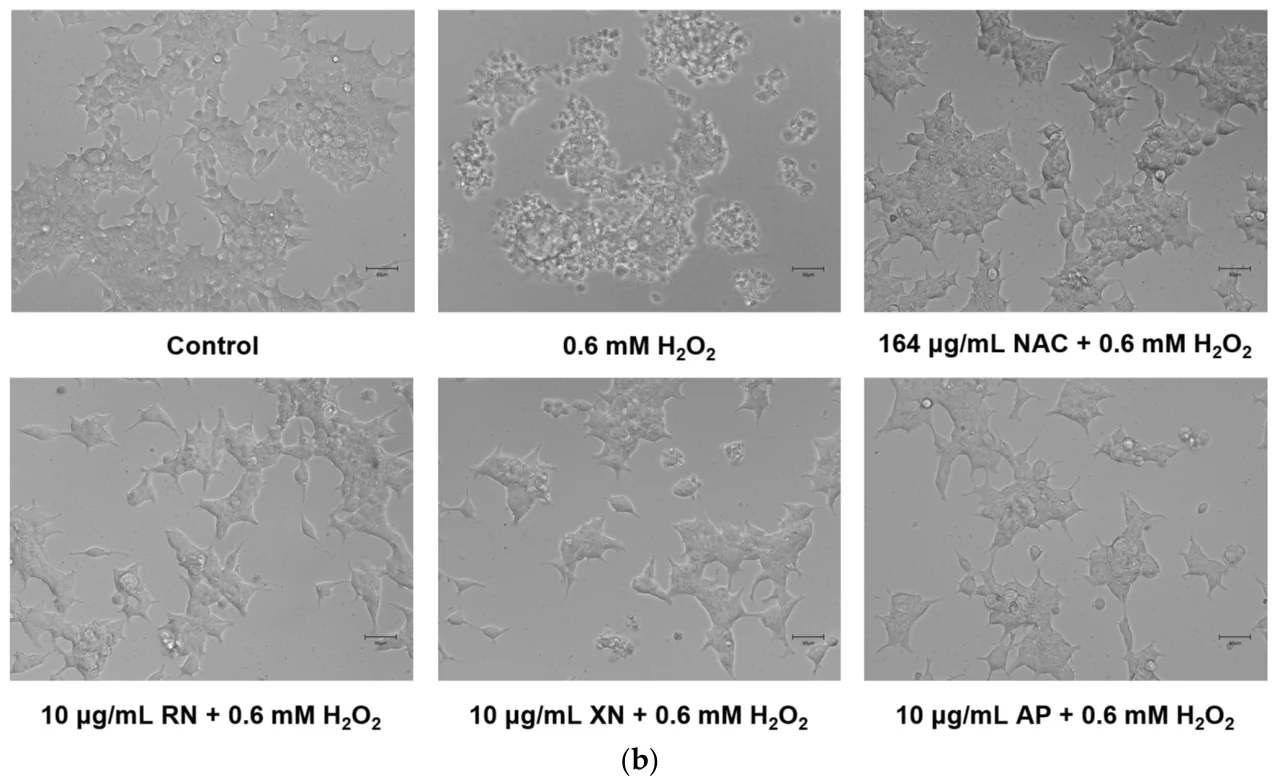
Protective effects of melanins and arginine-modified melanins (10 μg/mL) from different fungi on SH-SY5Y cell viability (**a**) and morphology (**b**) against 0.6 mM H_2_O_2_. Groups sharing different letters are significantly different (*p* < 0.05, Tukey’s post hoc test). Data shown are mean ± SD (n = 3 independent experiments performed in triplicate). **STM** and **ASTM**: synthetic melanin and modified synthetic melanin; **RN** and **ARN**: melanin and modified melanin from *R. nigricans*; **XN** and **AXN**: melanin and modified melanin from *X. nigripes*; **AP** and **AAP**: melanin and modified melanin from *A. pyriforme*; **SSsk** and **ASSsk**: melanin and modified melanin from skin of *S. sinnamariense*; **SSsp** and **ASSsp**: melanin and modified melanin from spore of *S. sinnamariense*. **NAC**: positive control; **white bar**: untreated cells/cells without H_2_O_2_; **black bar**: cells treated with H_2_O_2_ without sample.

The neuroprotective potential of the melanins and modified melanins against H_2_O_2_-induced cytotoxicity was assessed by incubating cells with 10 μg/mL of melanin/modified melanin for 24 h followed by a 24 h co-treatment with 0.6 mM H_2_O_2_. The positive control contained 164 μg/mL NAC, which is one of the most widely utilized neuroprotective antioxidants frequently serving as a positive control in neuroprotective studies and OS models [41,42]. The data for the screening are shown in Figure 2.

Figure 2a shows that 0.6 mM H_2_O_2_ reduced SH-SY5Y cell viability to 55.34% (negative control). This cytotoxicity was significantly attenuated by the administration of 10 μg/mL of melanins from *A. pyriforme*, *R. nigricans*, or *X. nigripes*, by which viability was increased to 75.31%, 74.60%, and 73.92%, respectively. These restored levels were comparable to the positive control (87.14%), thereby confirming the neuroprotective efficacy of these melanins against H_2_O_2_-induced injury on SH-SY5Y cells.

Morphological assessment by phase-contrast microscopy also supported the protective effect results (Figure 2b). Cells exposed to 0.6 mM H_2_O_2_ alone displayed characteristic features of neurotoxicity, including cell shrinkage and reduced cell density compared to untreated controls. In contrast, cells treated with melanins from *A. pyriforme*, *R. nigricans*, *X. nigripes*, and the positive control largely retained normal morphology, the same as that of the untreated cells, which included better cell attachment and density. However, some cells within these groups still exhibited an H_2_O_2_-induced effect.

In the following experiments, the neuroprotective effects of different concentrations of melanins from *A. pyriforme*, *R. nigricans*, and *X. nigripes* were further investigated.

### 2.4. Cell Protective Effects and ROS Assessment of Different Concentrations of Potent Melanins on SH-SY5Y Cells Against H_2_O_2_ Toxicity

Based on the initial screening at 10 μg/mL, potent melanins demonstrating significant neuroprotective effects were chosen for an expanded dose–response assay (2–20 μg/mL). Although the initial broad toxicity screening required a uniform concentration of 10 μg/mL to accommodate all samples safely (as some samples dropped below the 70% viability threshold at 20 μg/mL), the specific potent candidates selected for further testing had independently demonstrated excellent viability up to 20 μg/mL in the baseline cytotoxicity assay. Therefore, expanding the concentration range up to 20 μg/mL for these specific potent melanins allowed for the characterization of dose-dependent protective effects within the exact concentrations proven to be safe for them.

**Figure 3:**
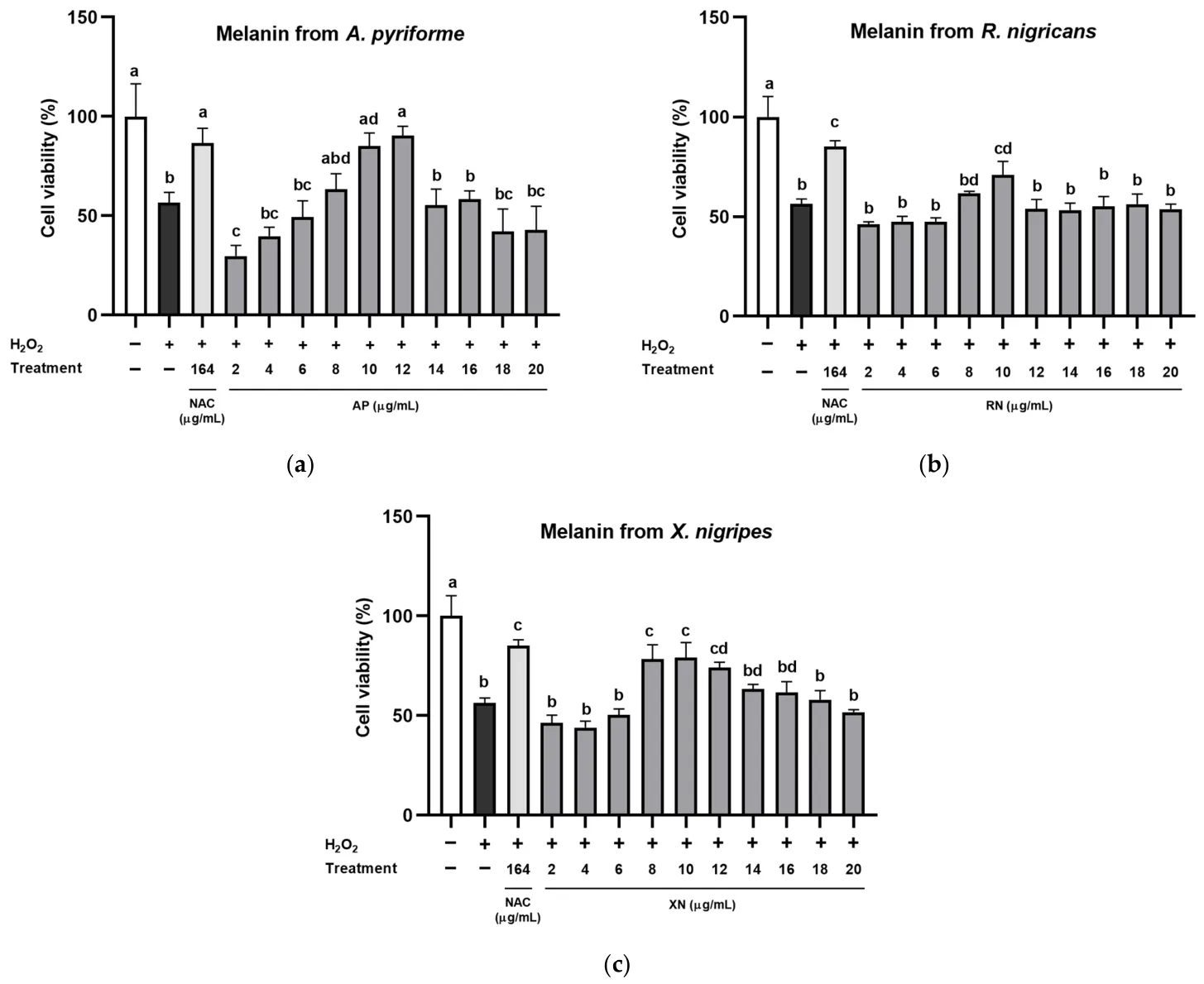
Protective effects of different concentrations of potent melanins including (**a**) melanins from *A. pyriforme* (**AP**), (**b**) melanins from *R. nigricans* (**RN**), and (**c**) melanins from *X. nigripes* (**XN**) against 0.6 mM H_2_O_2_-induced toxicity. Data shown are mean ± SD (n = 3 independent experiments performed in triplicate). Groups sharing different letters are significantly different (*p* < 0.05, Tukey’s post hoc test). **NAC**: positive control; **white bar**: untreated cells/cells without H_2_O_2_; **black bar**: cells treated with H_2_O_2_ without sample.

**Figure 4:**
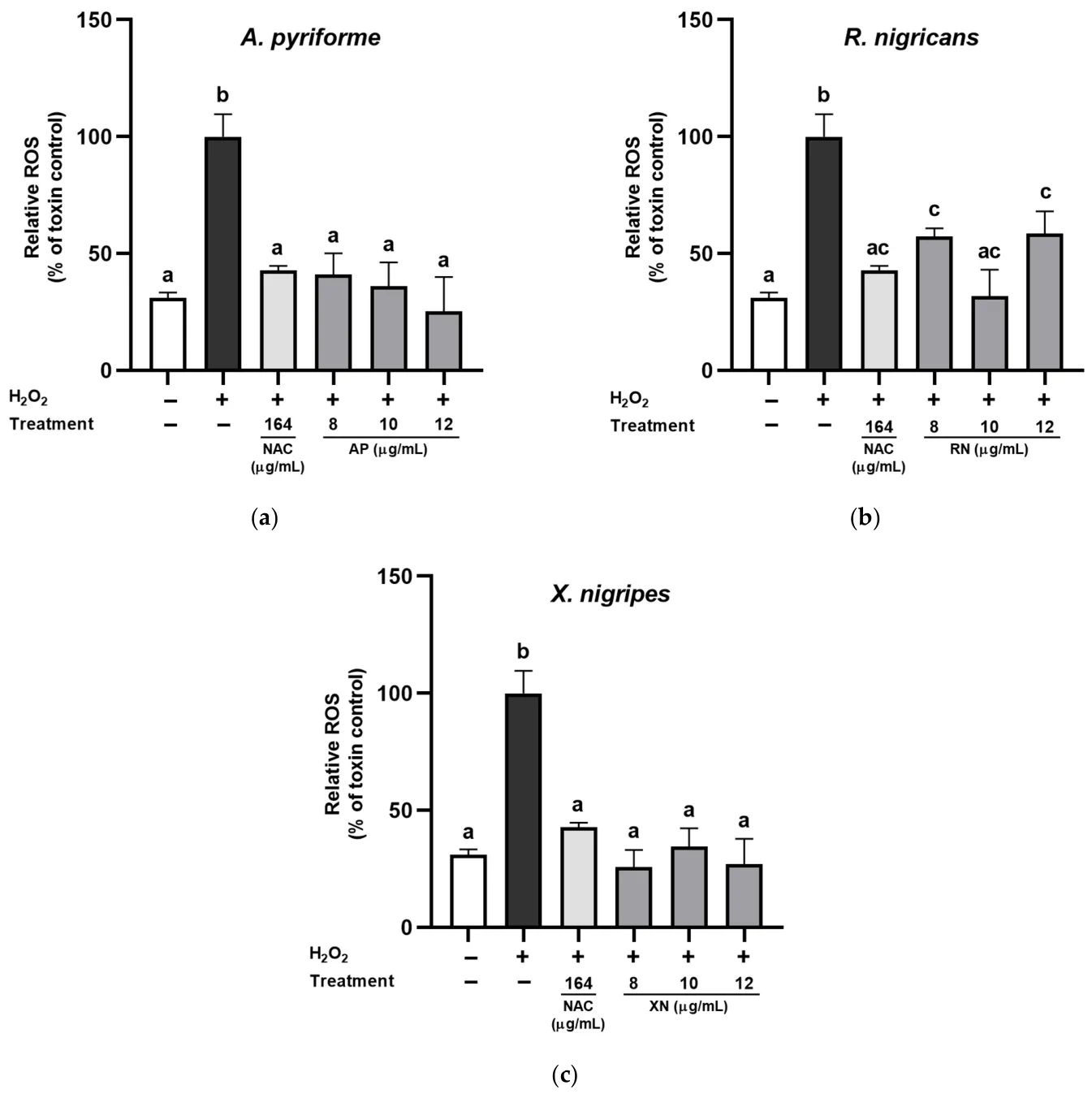
ROS assessment of different concentrations of potent melanins including (**a**) melanins from *A. pyriforme* (**AP**), (**b**) melanins from *R. nigricans* (**RN**), and (**c**) melanins from *X. nigripes* (**XN**) on SH-SY5Y cells against 0.6 mM H_2_O_2_. Groups sharing different letters are significantly different (*p* < 0.05, Tukey’s post hoc test). Data shown are mean ± SD (n = 3 independent experiments performed in triplicate). **NAC**: positive control; **white bar**: untreated cells/cells without H_2_O_2_; **black bar**: cells treated with H_2_O_2_ without sample.

The neuroprotective effects of melanins from *R. nigricans*, *X. nigripes*, and *A. pyriforme* against H_2_O_2_-induced toxicity were evaluated at different concentrations (2, 4, 6, 8, 10, 12, 14, 16, 18, and 20 μg/mL). The obtained data on neuroprotection are presented in Figure 3, and data on ROS measurements of the potent samples are presented in Figure 4.

The results showed that the melanins of the three samples all exhibited a relatively bell-shaped graph in which intermediate concentrations (8 to 12 μg/mL) provide maximal protection, while a mild protective effect was recorded at lower doses and higher doses lose efficacy or become toxic (Figure 3). This pattern is consistent with hormesis, whereby moderate doses induce adaptive antioxidant and cytoprotective responses sufficient to counteract OS, whereas excessive doses may disturb redox homeostasis and offset these beneficial effects, resulting in reduced neuroprotection [43].

In Figure 3 and Figure 4, among the tested concentrations, at 10 and 12 μg/mL, melanin from *A. pyriforme* displayed the most promising potential in restoring SH-SY5Y cell viability from 56.74% (negative control) to 85.05% and 90.32%, which are similar to the viability of the untreated cells and the NAC group (86.58%). This protective effect was further supported by ROS levels in these treatment groups (36.00% and 25.13%, respectively), which were statistically comparable (*p* > 0.05) to those of both the untreated control (30.89%) and the NAC-treated positive control group (42.80%). At 10 μg/mL, melanin from *R. nigricans* significantly increased cell viability from 56.36% to 70.93%. Although this recovery was statistically comparable to the NAC group (85.00%), it remained lower than that of both the untreated and positive control groups. Notably, this lower viability persisted despite robust ROS reduction (31.78%), which successfully matched the levels observed in the untreated (30.89%) and positive control (42.80%) groups. At concentrations of 8, 10, and 12 μg/mL, *X. nigripes* melanin exhibited a degree of protection resembling that of the NAC group (85.00%), increasing cell viability from 56.36% to 78.47%, 79.03%, and 74.04%, respectively. These cell viability values are significantly higher than the negative control (cells treated with H_2_O_2_) but lower than that of the untreated cells. However, ROS values of these treatments were 25.64%, 34.61%, and 27.11%, respectively, which are comparable to the ROS measured in the untreated control and the NAC group. It should be mentioned that the enhancement of cell viability in the above treatments seemed to relate to the reduction in ROS to the same value observed in the untreated cells. However, significant reduction in ROS, even to the same level found in the untreated cells, did not necessarily result in a significant enhancement of cell viability, as seen for melanins from *R. nigricans* at 8, 10, and 12 μg/mL and melanin from *A. pyriforme* at 8 μg/mL (Figure 4).

As previously reported, melanins from *A. pyriforme* (10 and 12 μg/mL) can effectively protect cells against H_2_O_2_ and maintain the same level of cell viability in the H_2_O_2_-treated cell as that of the untreated group and the positive control, suggesting that this melanin is a potent natural neuroprotective agent. In addition, our previous study found that the melanin from *A. pyriforme* is also safe for human kidney cells [38]. Therefore, it is a promising candidate for follow-up research in the development of complementary therapy for neuroprotection studies.

### 2.5. Screening for Potent Melanins and Arginine-Modified Melanins with Cell Protective Effects on SH-SY5Y Cells Against MPP^+^ Cytotoxicity

**Figure 5:**
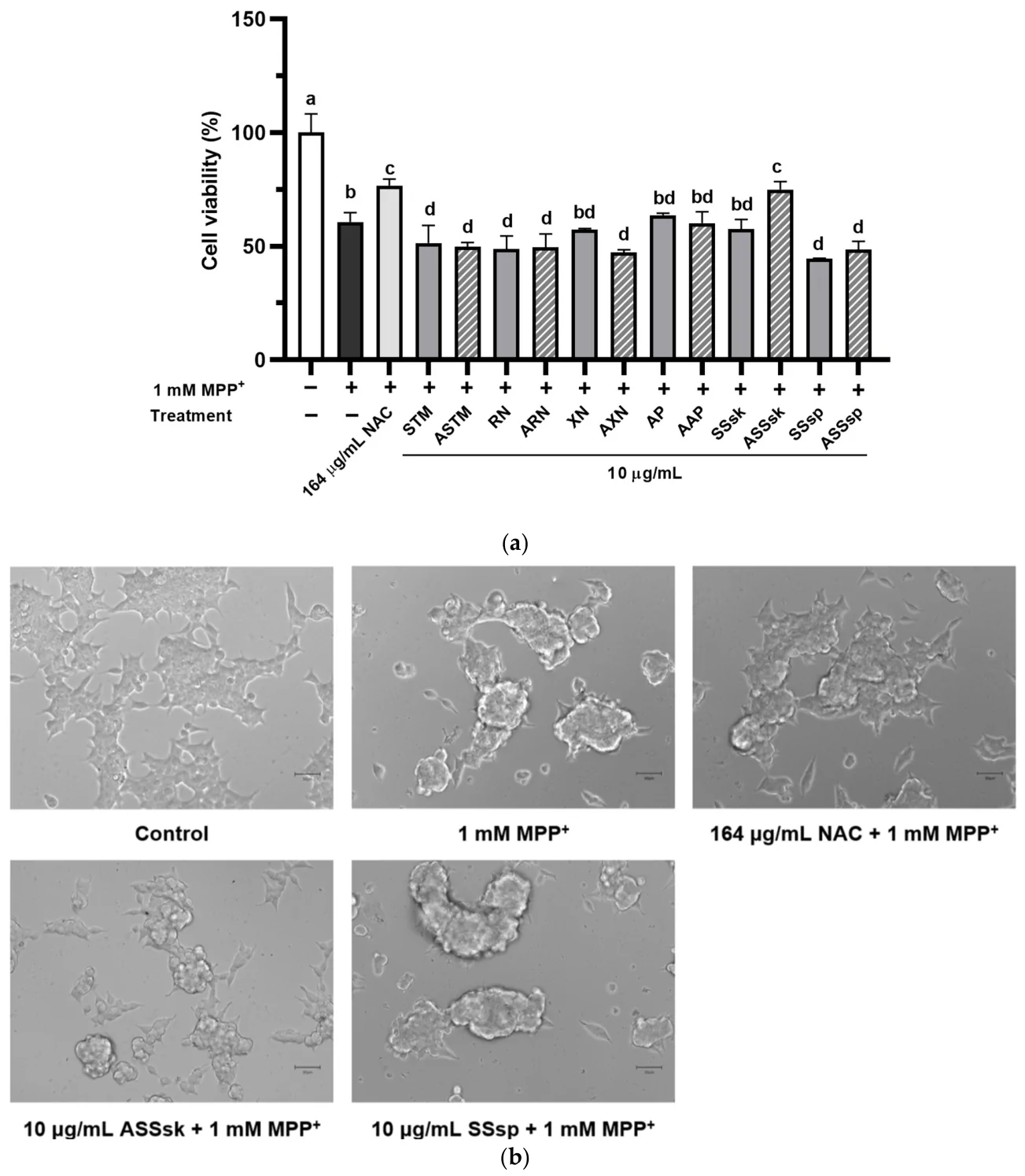
Protective effects of melanins and arginine-modified melanins (10 μg/mL) on SH-SY5Y cell viability (**a**) and morphology (**b**) against 1 mM MPP^+^. Groups sharing different letters are significantly different (*p* < 0.05, Tukey’s post hoc test). Data shown are mean ± SD (n = 3 independent experiments performed in triplicate). **STM** and **ASTM**: synthetic melanin and modified synthetic melanin; **RN** and **ARN**: melanin and modified melanin from *R. nigricans*; **XN** and **AXN**: melanin and modified melanin from *X. nigripes*; **AP** and **AAP**: melanin and modified melanin from *A. pyriforme*; **SSsk** and **ASSsk**: melanin and modified melanin from skin of *S. sinnamariense*; **SSsp** and **ASSsp**: melanin and modified melanin from spore of *S. sinnamariense*. **NAC**: positive control; **white bar**: untreated cells/cells without MPP^+^; **black bar**: cell treated with MPP^+^ without sample.

To evaluate the neuroprotective effect of melanins and arginine-modified melanins against the Parkinsonism toxin, the 0.6 mM H_2_O_2_ in the previous experiment was replaced with 1 mM MPP^+^, and the obtained data are presented in Figure 5.

According to Figure 5a, exposure to MPP^+^ reduced SH-SY5Y cell viability to 60.65%. At 10 μg/mL, among the tested samples, only arginine-modified melanin from *S. sinnamariense* (skin) significantly improved viability from 60.65% to 74.92%, and this activity was similar to that of the positive control (76.72%). However, both these values are significantly lower than those of the untreated group. Microscopic observations showed that normal cell morphologies were observed in both arginine-modified melanin from *S. sinnamariense* (skin) and the positive control, whereas cells in the MPP^+^-induced toxicity exhibited pronounced shrinkage and aggregation (Figure 5b).

### 2.6. Cell-Protective Effects and ROS Assessment of Different Concentrations of Arginine-Modified Melanin from S. Sinnamariense on SH-SY5Y Cells Against MPP^+^ Toxicity

**Figure 6:**
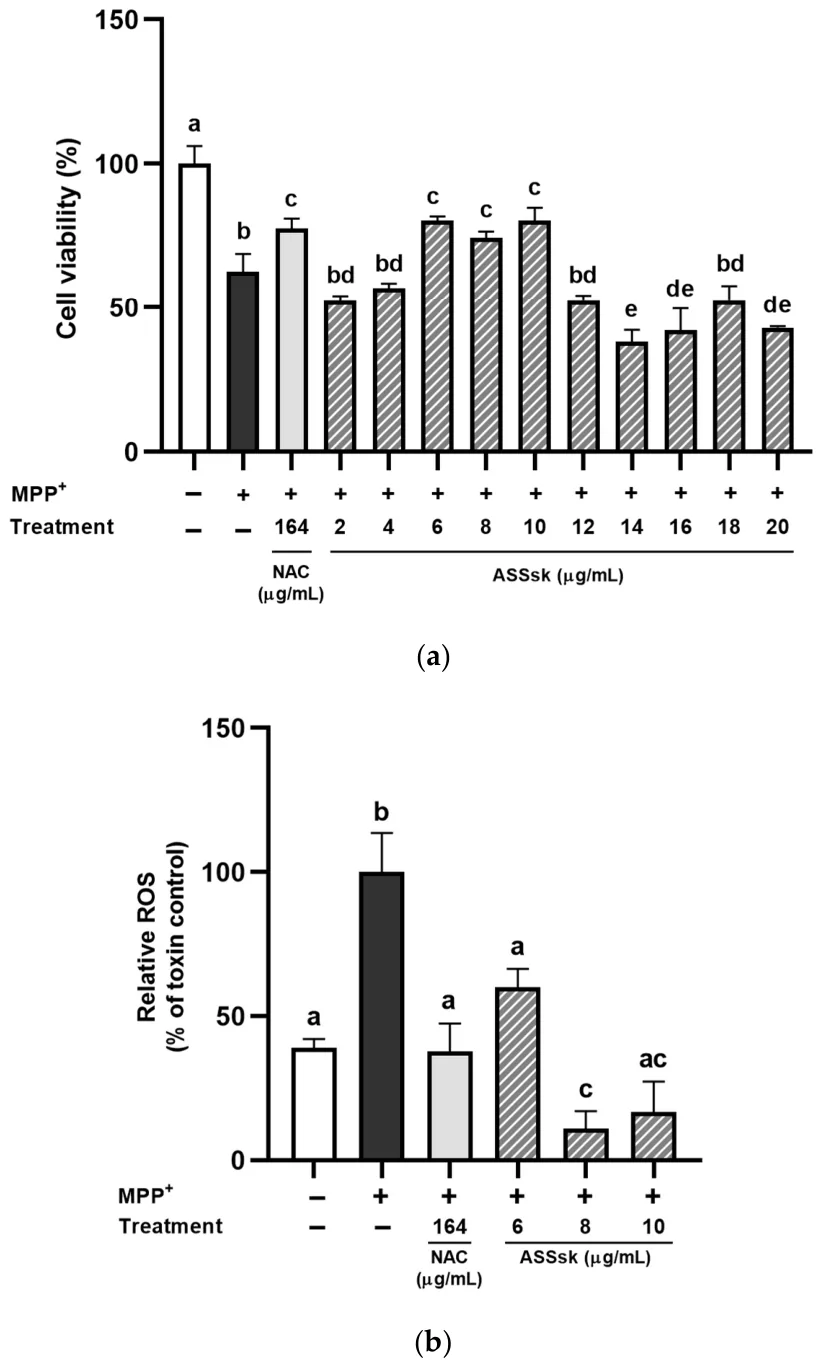
Protective effects of different concentrations of arginine-modified melanin from the skin of *S. sinnamariense* (**ASSsk**) on (**a**) SH-SY5Y viability against 1 mM MPP^+^ and (**b**) corresponding ROS level. Data shown are mean ± SD (n = 3 independent experiments performed in triplicate). Groups sharing different letters are significantly different (*p* < 0.05, Tukey’s post hoc test). **NAC**: positive control; **white bar**: untreated cells/cells without MPP^+^; **black bar**: cells treated with MPP^+^ without sample.

The neuroprotective effects of arginine-modified melanin from *S. sinnamariense* (skin) against MPP^+^-induced toxicity on SH-SY5Y cells was evaluated at different concentrations (2, 4, 6, 8, 10, 12, 14, 16, 18, and 20 μg/mL) and ROS measurement were conducted with the potential treatments. The obtained data are shown in Figure 6.

The protective effect of the sample was found to be most significant at concentrations of 6, 8, and 10 μg/mL, which enhanced cell viability from 62.62% to 80.42%, 74.50%, and 80.25%, respectively. These values are similar to those of the NAC group (77.71%), but significantly lower than the untreated cells. Concentrations below 6 μg/mL displayed no significant enhancement in cell viability, and concentrations above 12 μg/mL exhibited toxicity. Therefore, ROS evaluation was conducted with the treatments containing 6, 8, and 10 μg/mL of arginine-modified melanin from *S. sinnmariense*. The data revealed that these treatments significantly attenuated ROS levels in MPP^+^-exposed cells. At 6 μg/mL, the reduction (60.01%) was comparable to that of the NAC group. At higher concentrations (8 and 10 μg/mL), ROS levels dropped significantly lower (11.10% and 16.65%, respectively) than both the untreated control (39.04%) and the NAC group (37.69%). Interestingly, no statistical differences in cell viability were observed among these three concentrations, despite the significant variations in their respective ROS profiles.

Due to a dual protective potential exhibited against both toxins during the initial 10 μg/mL screening, *A. pyriforme* and *S. sinnamariense* were selected for further evaluation across an expanded concentration range of 2–20 μg/mL against MPP^+^ and H_2_O_2_ toxicity, respectively. Although arginine-modified melanin from skin of *S. sinnamariense* (at 6, 8, and 10 μg/mL) significantly attenuated MPP^+^-induced cytotoxicity, it showed no protective effect against H_2_O_2_-induced oxidative damage at any tested concentration (2–20 μg/mL) (Supplementary Figure S4). Conversely, treatment with *A. pyriforme* melanin (10 and 12 μg/mL) successfully mitigated H_2_O_2_ toxicity, yielding cell viability statistics equivalent to the untreated and positive controls. This protective capacity did not, however, extend to MPP^+^-treated cells (Supplementary Figure S5). In addition, the positive control also displayed better neuroprotective effect against H_2_O_2_ than MPP^+^ (Figure 2 and Figure 5).

## 3. Discussion

In this study, the extracted melanins consistently exhibited a black coloration across all studied fungal species, matching the characteristic appearance of fungal melanin types. Most of the species, including *A. pyriforme*, *A. asiaticus*, *S. sinnamariense*, *C. gigantea*, *D. tricolor*, *F. fomentarius*, and *R. nigricans* are basidiomycetes, which generally produce a dark brown to black eumelanin type [44,45]. This aligns with the visual observations of our extracted samples (Supplementary Figure S2). Meanwhile, *D. concentrica* and *X. nigripes* are ascomycetes that predominantly produce DHN melanin or pyomelanin; both pigments typically impart dark brown to black coloration, consistent with the actual colors observed in the melanins from these two fungi species [46].

The selection of potent fungal melanins for neuroprotective evaluation was guided by parameters including melanin yield, solvent solubility, and the reported biological properties (e.g., antioxidant activity). From a scalability perspective, production feasibility depends on both melanin content in the biomass and overall biomass yield, with the latter being directly governed by fruiting body size and abundance. However, a critical challenge in candidate selection is balancing yield against biomass accessibility, solvent compatibility, and other aspects. Although *F. fomentarius* provided the highest melanin yield in the initial screening, its poor solubility in 0.25 M NaOH presented a significant barrier to cell culture delivery. Similarly, *C. gigantea* and *D. concentrica* were excluded due to their resistance to arginine modification. Consequently, *A. pyriforme* and *R. nigricans* were selected due to their high yields, large and abundant fruiting bodies, optimal solvent compatibility, and chemical amendability to arginine modification. Although melanins from *X. nigripes* and both the skin and spore of *S. sinnamariense* exhibited lower extraction yields, *X. nigripes* is supported by its long-standing profile in traditional medicine [47], while *S. sinnamariense* is highly abundant in nature. In addition, melanins from these two species are also soluble in 0.25 M NaOH and can be easily modified with arginine. Furthermore, they also displayed significant antioxidant activity [40]. Therefore, these species including *A. pyriforme*, *R. nigricans*, *S. sinnamariense*, and *X. nigripes* were chosen for downstream biological assays.

Nevertheless, the extraction conditions also influence the production yield of melanin, and the optimal conditions vary across species due to the structural and chemical complexity of their respective fruiting bodies. The extraction protocols used in this study were not individually optimized for each species.

Following yield assessment and candidate selection, the comparative effects of these melanins were evaluated in SH-SY5Y cells. Variation in toxicity among melanins from different fungal species can be explained by their difference in structure and subsequently biological properties; different fungi produce distinct melanin types, and even species producing the same type of melanin do not necessarily yield identical polymeric structures [38,48].

Considering the highly pigmented color of melanin, melanins and arginine-modified melanins were tested in a cell-free culture system to exclude potential interference with the MTT compound. According to the results, the absorbance values of melanin and arginine-modified melanin did not differ from the blank (with usual cell culture medium only), even at the highest concentrations, indicating that interference of melanins with the MTT compound was negligible.

Because the MTT assay primarily reflects mitochondrial metabolic activity, decreases in MTT reduction may indicate metabolic impairment rather than irreversible cytotoxicity [49]. Accordingly, the concentration-dependent reduction observed at higher melanin concentrations may reflect metabolic suppression or other dose-dependent effects on cellular function. Furthermore, although melanins are generally regarded as antioxidants, exposure to high concentrations of melanins in a healthy cell system can disrupt cellular redox homeostasis and impair mitochondrial function, potentially shifting their effects toward pro-oxidant activity [50,51]. Hence, this concentration-dependent phenomenon may account for the progressive reduction in SH-SY5Y cell viability observed at higher melanin concentrations.

Fungal melanin can be modified using different amino acids, among which arginine is a common choice for many species [31,52,53,54]. Our previous study found that arginine modification of melanins significantly enhanced both the water solubility and antioxidant activity of melanins from various fungi; thus, arginine was used in this current study. Arginine-modified melanins generally displayed lower toxicity than their unmodified counterparts. Unlike melanins, the arginine-modified forms were water-soluble, allowing for preparation in water instead of NaOH. Consequently, this modification avoided the toxicity associated with high NaOH concentration in 100 μg/mL melanin solution. The improvement in water solubility is attributed to the nature of arginine as a strong cation, which may covalently bond with melanins by receiving their unpaired electrons through the binding of the carboxyl groups on melanin to the amine groups of arginine [31,55]. Previous studies have shown that amino acid modification can significantly alter the physicochemical properties of melanin, specifically enhancing its antioxidant activity and chemical behavior [38,52,53,54]. In addition, our initial zeta potential comparison of native melanin and arigine-modified melanin from *S. sinnamariense* showed that arigine modification significantly reduced the negative charge of melanin (unpublished data). This reduction in the surface charge may account for the difference in the effects of melanins and arginine-modified melanins on SH-SY5Y cells. However, it is not possible to point out the exact reasons within the scope of this study.

As arginine modification was performed by mixing melanin with an arginine solution at the designated ratio [31], a fraction of unreacted (unbound) arginine would possibly remain within the arginine-modified melanin matrix. Potential confounding effects of arginine in the arginine-modified melanin matrix on the cells were excluded by evaluating various concentrations of this amino acid, and no effects on cell viability were observed (Supplementary Figure S3).

While ROS are essential for normal neuronal signaling and cellular homeostasis, their overproduction can disrupt redox balance and contribute to neuronal dysfunction and neurodegeneration [7]. H_2_O_2_ is widely utilized for the induction of OS and ROS imbalance due to its ability to readily penetrate cellular membranes, and it takes part in generating several ROS, such as highly reactive hydroxyl radicals (OH^●−^), resulting in oxidative damage to lipids, proteins, and nucleic acids [51]. Once the safe concentration thresholds for both melanins and arginine-modified melanins were established, their protective effects against H_2_O_2_-induced OS were investigated. In the present study, the most potent protective effects among all tested fungal melanins were exhibited by the melanins obtained from *A. pyriforme*, *R. nigricans*, and *X. nigripes* at 10 μg/mL. The potent protective effects of *R. nigricans* and *X. nigripes* also exhibit clear congruence with our previous findings regarding their strong ABTS radical-scavenging activities [38].

Based on this preliminary screening, these melanins were selected for an expanded dose–response evaluation. The experimental range from 2 to 20 μg/mL for selected candidates was dictated by a balance between broad screening rigor and individual sample tolerability. In the initial evaluation across all extracted and modified melanins, 10 μg/mL was established as the maximum uniform baseline concentration, as it consistently maintained cell viability above 70% across nearly all initial fungal samples. While a concentration of 20 μg/mL induced mild toxicity in a few samples, melanins from *A. pyriforme*, *R. nigricans*, and *X. nigripes* as well as the skin of *S. sinnamariense* (the potent sample against MPP^+^ toxicity) had demonstrated no toxicity at this higher dose during the screening. Consequently, testing these specific potent candidates up to 20 μg/mL was fully justified. This approach permitted a thorough exploration of the dose–response dynamics of these promising strains, maximizing the resolution of the cytoprotective data against H_2_O_2_ and MPP^+^ toxicity within their safe dosing limits.

The ROS assessment on the protective effects of potent melanins (*A. pyriforme*, *R. nigricans*, and *X. nigripes*) against H_2_O_2_ toxicity suggests that, although some melanins can attenuate ROS accumulation at the tested concentrations, ROS reduction does not always necessarily correspond to cell viability recovery, as additional factors such as mitochondrial dysfunction or downstream cellular damage may limit the recovery of cell viability. As previously mentioned, as MTT assay primarily reflects mitochondrial metabolic activity, it is possible that mitochondrial dysfunction or other downstream damage might persist despite the reduction in ROS levels in these cases. Although increased cell viability indicates protection against toxin-induced damage, it does not identify the exact mechanism involved.

Although DCFH-DA is frequently used to detect intracellular ROS, this probe measures general intracellular ROS and does not directly react with H_2_O_2_ at an appreciable rate [56]. Instead, the oxidation of DCFH to the highly fluorescent DCF requires the presence of a variety of secondary, more potent oxidants, for example, hydroxyl radicals (OH^●−^), which are typically generated when interacting with redox-active metal ions (like iron or copper) or heme proteins via the Fenton reaction [56]. The reduction in intracellular ROS levels assessed by the DCFH-DA probe is likely due to the antioxidant properties of melanin. Melanin can directly scavenge ROS through its redox-active functional groups and stable unpaired electrons [11] while simultaneously chelating transition metal ions that catalyze Fenton reactions. This limits the conversion of H_2_O_2_ into highly reactive hydroxyl radicals (OH^●−^) and directly neutralizes any remaining radicals before they can oxidize the DCFH probe [11,19]. Consequently, the decrease in measured fluorescence observed in H_2_O_2_-treated cells may represent melanin’s ability to disrupt the metal-dependent pathways that produce the specific oxidants required to activate the DCFH signal.

Another point worth considering is that after the pretreatment (where cells were incubated with melanin/arginine-modified melanin alone), the culture medium was completely removed, likely eliminating non-internalized melanins while retaining internalized melanins that safely sequester intracellular metal ions. During the subsequent 24 h co-treatment, fresh medium containing melanin/modified melanin alongside the toxin (H_2_O_2_) was added to the cells and incubated for an additional 24 h. Consequently, part of the observed neuroprotection may originate from a direct interaction between the melanin present outside the cells and H_2_O_2_. Previous studies suggest that melanin possesses a high capacity to catalyze the decomposition of H_2_O_2_ [57], thereby mitigating H_2_O_2_-induced toxicity and reducing the substrate pool available for intracellular Fenton reactions with transition metals or heme proteins. Taken together, melanin likely exerts neuroprotection through dual intracellular (metal sequestration) and extracellular (H_2_O_2_ catalytic breakdown) actions. However, further studies are required to elucidate the precise underlying mechanisms.

Unlike H_2_O_2_, which primarily causes oxidative damage through ROS generation, MPP^+^, the toxic metabolite formed from MPTP (1-methyl-4-phenyl-1,2,3,6-tetrahydropyridine), exerts its neurotoxicity by selectively accumulating in dopaminergic neurons and inhibiting mitochondrial complex I of the electron transport chain after entering the brain [58]. This inhibition impairs ATP production, promotes mitochondrial dysfunction, and triggers secondary ROS generation, ultimately leading to neuronal death [58]. The differences in the protective effects of the samples observed between the H_2_O_2_ and MPP^+^ treatments suggest that the toxicity mechanisms of these two toxins, as well as the properties of melanin/modified melanin samples, are distinct. These mechanistic distinctions are further corroborated by the cellular morphological variations presented in Figure 2 and Figure 5.

Most melanins and modified melanins, even those with H_2_O_2_-protective potential, showed no significant protective effects on SH-SY5Y cells against MPP^+^ damage. Only the arginine-modified melanin from *S. sinnamariense* exhibited significant protection against MPP^+^-induced toxicity, despite demonstrating only a weak protective effect against H_2_O_2_ toxicity. This highly selective activity suggests that different mechanisms might mediate the protective actions of fungal melanins against H_2_O_2_ and MPP^+^, driven primarily by species-dependent structural variations. The protective effect of arginine-modified melanin of *S. sinnamariense* against MPP^+^ may not only require the antioxidant capacity of melanins but could also suggest a greater ability to mitigate mitochondrial damage, to attenuate secondary ROS generation, or to interact with MPP^+^-associated toxic pathways, although the precise mechanism remains to be elucidated. Regarding assay specificity, the DCFH-DA probe may detect the ROS generated downstream of MPP^+^-induced mitochondrial dysfunction, therefore reflecting the accumulation of oxidizing species such as hydroxyl radicals (OH^●−^), peroxynitrite (ONOO^−^), and hydroperoxides (^−^OOH), which readily oxidize DCFH into fluorescent DCF [59].

Another possible explanation is that arginine modification of melanin altered its physicochemical characteristics, as previously mentioned, possibly resulting in enhanced protection against MPP^+^-induced neurotoxicity. More importantly, arginine alone did not significantly improve cell viability in the MPP^+^ model at concentrations equivalent to those present in the modified melanin treatments (Supplementary Figure S6), indicating that the neuroprotective effect was associated with the arginine-modified melanin rather than arginine itself. However, as current evaluations reflect general intracellular ROS accumulation rather than specific radical species, it is not possible to pinpoint the exact protective mechanisms of the above samples. Consequently, further studies assessing mitochondrial membrane potential, caspase cascades, or specific downstream biomarkers of macromolecular injury, such as lipid peroxidation and protein oxidation, would be the right next steps to clarify these mechanisms. In addition, the findings from both the present work and our previous study indicate that melanin samples and their derivatives possess distinct structural and physicochemical properties; subsequently, they display distinct cytoprotective profiles against different toxins (H_2_O_2_ and MPP^+^).

Although the melanins were extracted and purified using well-established protocols, due to the absence of membrane dialysis during the purification process, the samples may retain residual small-molecule impurities and inorganic salts from the acid–base precipitation treatments. If present, these residual species would represent potential confounding factors; specifically, unremoved salts may alter the ionic strength of the medium while subtly affecting localized osmotic dynamics during cell-based assays [60]. While background-subtraction protocols and cell-washing steps were applied to mitigate optical interference in the used assays, incorporating membrane dialysis to ensure complete impurity removal should be periodized in future studies.

## 4. Materials and Methods

### 4.1. Materials

Potassium hydroxide (KOH, Cat. No. 1310-58-3) and hydrochloric acid 37% (HCl, Cat. No. 7647-01-0) were purchased from Xilong Scientific (Shantou, China). Absolute ethanol (Cat. No. 64-17-5) and chloroform (Cat. No. 67-66-3) were obtained from Chemsol (Ho Chi Minh City, Vietnam). SH-SY5Y and neuroblastoma cells (CRL-2266™) were purchased from the American Type Culture Collection (ATCC) (Manassas, VA, USA). Cell Proliferation Kit I (Cat. No. 11465007001, Mannheim, Germany) was purchased from Roche. 2′,7′-dichlorofluorescin diacetate (DCFH-DA, Cat. No. D6883, Rehovot, Israel), Dimethyl sulfoxide (DMSO, Cat. No. D4540, St. Gallen, Switzerland), 1-Methyl-4-phenylpyridinium (MPP^+^) iodide (Cat. No. D048, Shanghai, China), N-Acetyl-L-Cysteine (NAC, Cat. No. A7250, Shanghai, China), penicillin/streptomycin 1% (Cat. No. P4333) were purchased from Sigma Aldrich Co., LLC (St. Louis, MO, USA). Gibco Fetal Bovine Serum (FBS, Cat. No. 10270-106, Brazil origin), Gibco Hank’s Balanced Salt Solution (1×) (HBSS, Cat. No. 14025-092), Gibco 0.05% Trypsin-EDTA (1×) (Cat. No. 25300-062), Gibco Phosphate-Buffered Saline (10×) (PBS, Cat. No. 7001-044), and hydrogen peroxide (H_2_O_2_) 35 wt.% solution (Cat. No. 202465000) were purchased from Thermo Fisher Scientific (Waltham, MA, USA). Dulbecco’s Modification of Eagle’s Medium (1×) (DMEM, Cat. No. 10-013-CV) was purchased from Corning Life Sciences (Manassas, VA, USA). All reagents used were of analytical, GC, or HPLC grade. Reagents for cell culture were all sterile filtered.

### 4.2. Methods

#### 4.2.1. Collection and Identification of Melanin-Producing Fungal Species

Mature fruiting bodies of different fungal species were collected from forests in Vietnam and Belgium between 2022 and 2024; sclerotia of *X. nigripes* were purchased from a local vendor in Ho Chi Minh city, Vietnam. Fruiting bodies of *S. sinnamariense* and *A. asiaticus* were collected around July, during the rain seasons in Vietnam, whereas Belgian specimens were collected through June–December.

Except for *X. nigripes* and *D. concentrica*, which were commercially purchased or previously identified, the fungal specimens were identified by sequencing the internal transcribed spacer (ITS) region. The ITS region was amplified with primers ITS1 (5′-CTTGGTCATTTAGAGGAAGTAA-3′) and ITS4 (5′-TCCTCCGCTTATTGATATGC-3′) [37]. The PCR products were analyzed by 1st BASE Laboratories (Malaysia) using Sanger sequencing. The obtained sequences were compared with reference sequences in the NCBI GenBank database using the BLAST search (http://blast.ncbi.nlm.nih.gov/).

#### 4.2.2. Melanin Extraction and Melanin Modification

Fungal samples were oven-dried at 70 °C and ground into fine powder. Melanin extraction and purification was performed according to Olaizola, 2012 and Lai et al., 2026 with some modifications [38,61]. One gram of the fungal powder was submerged into 60 mL of 1 M KOH and incubated for 3 h in a water bath at 100 °C. The mixture was then centrifugated at 11,000× *g* for 15 min at 25 °C to collect the supernatant. The pH value of the supernatant was adjusted to 2.0 by adding 2 M HCl, and then it was set aside at room temperature for 1 h for the precipitation of the crude pigment. The crude pigment was then collected by centrifugation at 11,000× *g* for 15 min at 25 °C, rinsed thrice with deionized water, and freeze-dried.

For purification, the freeze-dried crude powder was suspended in 15 mL of 7 M HCl solution and incubated at 100 °C for 2 h to hydrolyze acid-soluble impurities. The precipitate was then recovered by centrifugation at 11,000× *g* for 10–15 min at 25 °C, and washed once with chloroform, once with absolute ethanol, and twice with deionized water. Subsequently, the purified melanin was freeze-dried and stored at 4 °C for later use.

The extraction yield (amount of purified melanin obtained from one gram of fungal biomass) was calculated using the following formula:

Extraction yield (mg g^−1^) = M_M_/M_B_,

where M_M_ is the mass of purified melanin (mg), and M_B_ the weighed biomass (g).

The initial characterization of the extracted melanin from different fungal species was confirmed and structurally characterized using spectroscopic and Fourier-transform infrared resonance (FT-IR) analyses before subsequent modification [38].

Arginine modification of melanin was performed following Lai, Ngoc et al. (2026) [38]. Arginine was prepared at a specific concentration in sterile distilled water before being mixed and stirred with melanin at room temperature. The reaction was carried out using melanin-to-arginine ratios (*w*/*w*) of 1:2 for *A. pyriforme*, 1:1 for *R. nigricans*, and 1:5 for *S. sinnamariense* (skin), *S. sinnamariense* (spore), and *X. nigripes* [38].

#### 4.2.3. Cell Culture

The undifferentiated human neuroblastoma cell line (SH-SY5Y) was cultured in Dulbecco’s Modified Eagle’s Medium (DMEM) supplemented with 10% (*v*/*v*) fetal bovine serum (FBS) and 1% (*v*/*v*) penicillin/streptomycin (100 IU/mL and 100 µg/mL, respectively) according to the manufacturer’s protocol and maintained at 37 °C with 95% humidified air of 5% CO_2_. Cells were passaged at 80–90% confluency.

#### 4.2.4. Effects of Different Concentrations of Melanin and Modified Melanins on SH-SY5Y Cell Viability

Stock solutions of melanin and modified melanin (10 mg/mL) were prepared in 0.25 M NaOH and distilled water, respectively. Final working concentrations (10, 20, 40, 60, 80, and 100 μg/mL) were subsequently prepared by diluting the stock solution in DMEM supplemented with 1% FBS. Cells were seeded in 96-well plates at a density of 1 × 10^4^ cells/well, cultured for 24 h, and then treated with melanin or modified melanin at a certain concentration for another 24 h.

As the stock solution of melanin was prepared in 0.25 M NaOH, the NaOH concentration in each melanin treatment is dependent on the working concentration of melanin. More specifically, 250, 500, 1000, 1500, 2000, and 2500 μM NaOH was present in the cultures treated with 10, 20, 40, 60, 80, and 100 μg/mL of melanin, respectively. Therefore, these concentrations were included as vehicle controls for the corresponding melanin treatments. Melanin- and modified-melanin-only controls (without cells) were also included to subtract the potential background interference. The SH-SY5Y cell viability was then assessed via the MTT assay.

The MTT assay assesses cell viability by measuring the ability of living cells to reduce yellow MTT (3-(4,5-dimethylthiazol-2-yl)-2,5-diphenyltetrazolium bromide) into purple insoluble formazan crystals [49]. Following treatments, 10 μL of MTT stock solution (5 mg/mL) was added to each well containing 90 μL of DMEM, yielding a final concentration of 0.5 mg/mL in a total volume of 100 μL. After 4 h incubation, the formazan produced in SH-SY5Y cells was dissolved in 100 μL solubilization buffer (10% SDS in 0.01 M HCl) following MilliporeSigma’s protocol. The amount of MTT formazan product was determined by measuring absorbance at 570 nm with a 750 nm reference wavelength using a BioTek Synergy HT microplate reader (Winooski, VT, USA) [49]. The viability of SH-SY5Y cells was expressed as the percentage (%) of viable cells relative to the negative control (untreated cells) (100%).

Based on the cell viability results, melanin and modified melanin samples at a concentration of 10 μg/mL were selected for the protective effect evaluation as this concentration maintained cell viability above 70% across most samples and/or was comparable to the untreated controls.

#### 4.2.5. Cell Protective Effects of Melanins and Modified Melanins on SH-SY5Y Cells

Cells were plated in 96-well plates at a density of 1 × 10^4^ cells/well and were incubated overnight for attachment. The cells were then pre-treated with either melanin or modified melanin (10 μg/mL) for 24 h. The medium was then replaced with fresh medium containing 0.6 mM H_2_O_2_ (or 1 mM MPP^+^) in combination with melanin or modified melanin (10 μg/mL), and co-incubated for another 24 h (100 μL final total working volume per well). These concentrations of the toxins were selected because they reduced the cell viability to 55% and 60%, respectively. These values fall within the standard threshold for toxin selection in neuroprotection studies, as this level of toxicity preserves a larger population of viable cells while still providing a sufficient toxic challenge to evaluate the protective effects of the tested compounds [62,63].

The positive control contained 164 μg/mL N-Acetylcysteine (NAC), the negative control consisted of cells treated with the toxin but without melanin or modified melanin, and the untreated cells contained cells without toxin, melanin, or modified melanin. The cell viability was evaluated by the MTT assay and compared between the treatments, the negative control, and the untreated cells. Based on this result, potent melanin or modified melanin was selected for further evaluation of its neuroprotective effects at different concentrations (2–20 μg/mL). Subsequently, concentrations that displayed significant activities were selected for intracellular ROS assessment.

#### 4.2.6. Measurement of Intracellular ROS

Intracellular ROS was measured using 2′,7′-dichlorofluorescin diacetate (DCFH-DA), a cell-permeable fluorescent probe. After entering cells, DCFH-DA is deacetylated by intracellular esterases to form a non-fluorescent intermediate, which is subsequently oxidized by ROS into the fluorescent 2′,7′-dichlorofluorescein (DCF). The DCF fluorescence signal reflects the amount of oxidized DCF, which is proportional to the intracellular ROS level [64].

After treatments with melanin or modified melanin, followed by the toxin as described above, cells were washed with Hank’s Balanced Salt Solution (HBSS) and incubated with 10 μM DCFH-DA in HBSS for 30 min at 37 °C in the dark. After incubation, cells were washed twice with HBSS to remove the excess dye and resuspended in HBSS before fluorescence reading. Fluorescence intensity was measured at an excitation/emission wavelength of 504/529 nm using a Thermo Scientific Varioskan LUX microplate reader. To exclude the possible interference of melanins, cell-free melanin controls were included to evaluate baseline fluorescence effects. Following subtraction of cell-free background fluorescence, DCF fluorescence was normalized to the MTT absorbance obtained from corresponding parallel wells to account for treatment-associated differences in cell viability. The normalized DCF fluorescence ratio for each group was expressed as a percentage of the toxin-treated positive control group (H_2_O_2_ or MPP^+^ alone), which was set as 100%. The MTT-normalized DCFH-DA signal for each sample was calculated as below:

$${\mathrm{DCF}}_{\mathrm{norm}}=\left(\frac{{\mathrm{DCF}}_{\mathrm{fluoresence}}}{{\mathrm{MTT}}_{\mathrm{absorbance}}}\right)\times 100$$

Then, each condition relative to the toxin-treated positive control was expressed as follows:

$$\mathrm{Relative}\;\mathrm{ROS}\;(\%\;\mathrm{toxin}\;\mathrm{control})=\left(\frac{\mathrm{DCFnorm}}{\mathrm{DCFnorm}\;\mathrm{of}\;\mathrm{toxin}}\right)\times 100$$

#### 4.2.7. Statistical Analysis

All experiments were conducted in triplicate. Data are presented as the mean ± standard deviation (SD). Statistical significance among experimental groups was assessed by one-way analysis of variance (ANOVA) followed by Dunnett’s or Tukey’s multiple comparison tests, with statistical significance defined as *p* < 0.05. Dunnett’s multiple comparison test was employed for comparisons between the control group and the remaining experimental groups, whereas Tukey’s post hoc test was utilized for pairwise comparisons among all experimental groups.

## 5. Conclusions

In the present study, nine melanin-producing fungal species were successfully isolated and taxonomically verified. The yields of melanins significantly varied, with *A. pyriforme*, *C. gigantea*, *F. fomentarius*, and *R. nigricans* possessing the highest yields, ranging from 76.91 to 99.83 mg/g. However, *A. pyriforme*, *R. nigricans*, *X. nigripes*, and *S. sinnamariense* were selected for further evaluation based on a combination of their biomass availability, optimal alkaline solubility, ease of chemical modification with arginine, and previously reported antioxidant activity.

The neuroprotective effects of these five selected fungal melanins and their arginine-modified melanins against H_2_O_2_ and MPP^+^ toxicity were evaluated. Melanins from *A. pyriforme*, *R. nigricans*, and *X. nigripes* were found to be the most promising agents against H_2_O_2_. In contrast, only modified melanin from *S. sinnamariense* effectively protected cells against MPP^+^-induced cytotoxicity. These samples were further tested with various concentrations and their neuroprotective effects against the respective toxins were found to be dose dependent. At 10 and 12 μg/mL, melanin from *A. pyriforme* improved the viability of H_2_O_2_-treated cells to 85% and 90% and reduced the ROS levels to 36% and 25%, respectively. These values are similar to those of the toxin-free cells and the positive control.

Regarding MPP^+^-induced cytotoxicity, arginine-modified melanin from *S. sinnamariense* at 6, 8, and 10 μg/mL effectively protected cells. Both cell viability enhancement and ROS reduction values were comparable to those of the positive control, but significantly lower than those of the untreated cells.

In conclusion, among all fungal species, *A. pyriforme* provided a high yield of melanin with notable cytoprotective activity against H_2_O_2_-induced toxicity in SH-SY5Y cells. Therefore, this melanin sample represents a promising candidate for future in vivo PD research.

## Figures and Tables

**Table 1:** List of melanin-producing fungal species with their melanin yield and observed coloration.

Fungal Species	Color of Fruiting Body	Extraction Yield (mg g^−1^)	Color of Purified Melanin
*Apioderdon pyriforme*	brown	90.16 ± 2.41 ^a^	black
*Astraeus asiaticus*	brown	16.57 ± 1.57 ^b^	black
*Calvatia gigantea*	brown	76.91 ± 3.40 ^c^	black
*Daedaleopsis tricolor*	brown, reddish brown	10.74 ± 1.21 ^b^	black
*Daldinia concentrica*	brownish black	33.10 ± 2.84 ^d^	black
*Fomes fomentarius*	brown	99.83 ± 3.93 ^e^	black
*Russula nigricans*	black	78.65 ± 2.56 ^c^	black
*Scleroderma sinnamariense* (skin/peridium)	yellow	6.72 ± 0.12 ^bf^	black
*Scleroderma sinnamariense* (spore)	black	34.39 ± 1.99 ^d^	black
*X. nigripes*	black	3.46 ± 1.41 ^f^	black

Different letters (a to f) indicate a significant difference in melanin extraction yield between fungal species (*p* < 0.05, Tukey’s post hoc test). Data shown are mean ± SD (n = 3 independent experiments performed in triplicate). Comparisons were performed across all species to identify statistically distinct high- and low-yielding melanin-producing fungal species.

## Data Availability

The original contributions presented in this study are included in the article/Supplementary Material. Further inquiries can be directed to the corresponding authors.

## Supplementary Materials

The following supporting information can be downloaded at: https://www.mdpi.com/article/10.3390/molecules31173135/s1, Table S1: BLAST identification of ITS sequences of the melanin producing fungi; Table S2: ITS sequences of fungal species; Figure S1: Fruiting bodies of different melanin-producing fungi. (a). *Apioperdon pyriforme*; (b). *Astraeus asiaticus*; (c). *Calvatia gigantea*; (d). *Daedaleopsis tricolor*; (e). *Daldania concentrica*; (f). *Fomes fomentarius*; (g). *Russula nigricans*; (h). *Scleroderma sinnamariense*; (i). *Xylaria nigripes*; Figure S2: Melanin pigments from different fungal species. (a). *Apioperdon pyriforme*; (b). *Astraeus asiaticus*; (c). *Calvatia gigantea*; (d). *Daedaleopsis tricolor*; (e). *Daldania concentrica*; (f). *Fomes fomentarius*; (g). *Russula nigricans*; (h). *Scleroderma sinnamariense*; (i). *Xylaria nigripes*; Figure S3: Effects of different concentrations of arginine on SH-SY5Y cell viability. Different concentrations of arginine were selected based on the melanin:arginine (*w*/*w*) ratios used for the arginine modification of melanin and the tested concentrations of melanin and modified melanin on SH-SY5Y cells; Figure S4: Protective effects of different concentrations of arginine-modified melanin from skin of *S. sinnamariense* (ASSsk) against 0.6 mM H_2_O_2_; Figure S5: Protective effects of different concentrations of melanin from *A. pyriforme* (AP) against 1 mM MPP^+^; and Figure S6: Effects of arginine on cell viability of MPP+-induced SH-SY5Y cells.
