# Supplementary material for: Fungal Melanins as Potential Reactive Oxygen Species-Scavenging Neuroprotective Agents
Source: Molecules. 2026 Sep 7;31(17):3135. doi: 10.3390/molecules31173135 (PMC13567587; doi:10.3390/molecules31173135)
Supplement: Supplementary file 1 [file molecules-31-03135-s001.zip › molecules-4521191-supplementary.pdf]

Supplementary Materials

Supplementary Table S1. BLAST identification of ITS sequences of the melanin producing fungi.

Fungal samples	Habitat	Query cover	Percent Identity	Accession
Apioperdon pyriforme	Brussels, Belgium	100%	100%	AB811851.1, AY854075.1, KP454030.1, MN992465.1
Astraeus asiaticus	Dong Nai, Viet Nam	96%	99.23%	NR_119468.1
Calvatia gigantea	Brussels, Belgium	100%	99-100%	MW732168.1, LN714562.1, MW488327.1
Daedaleopsis tricolor	Brussels, Belgium	100%	100%	OK055706.1
Fomes fomentarius	Brussels, Belgium	100%	99.5-100%	MN065451, X290073, OL764369

Russula nigricans	Brussels, Belgium	98-99%	97-98%	AY061695, MW172333, EF126733
Scleroderma sinnamariense	Lam Dong, Viet Nam	100%	100%	LC651046.1

Supplementary Table S2. ITS sequences of fungal species

Apioperdon pyriforme	AGTTGTAGCTGGCTCTTTGGAGCATGTGCACGCTTGTCTTGATCTTATTCATCCACCTGTGCACATTTTGTAGTCTTTTGGG CTGAGTCGAGTATCGGATGGATGTCTATCCGGATGTGAGGACTGCTGAGTGCGAAAGCATACGGCTCTTCTCTGTTTGAC CTCACCCTTGAGTACTATGTATTCATATACCACATAGTATGTTGTAGAATGTGATCATTGGGCCTTTGTGCCTATAATAAT CATATACAACTTTCAGCAACGGATCTCTTGGCTCTCGCATCGATGAAGAACGCAGCGAAATGCGATAAGTAATGTGAAT TGCAGAATTCAGTGAATCATCGAATCTTTGAACGCACCTTGCGCTCCTTGGTATTCCGAGGAGCATGCCTGTTTGAGTGT CATTAAATTCTCAACCCCACTAGCTTTTGTGAGCTATGCTGGGGCTTGGATGTTGGGAGTTTGCGGGCCTTTACTAGGTC GGCTCTCCTGAAATACATTAGCGGAACCGTTTGCAGTCCCGTCACTAGTGTGATAATTATCTACACTGTGATGACTGCTC TCTGACTAGTTCAGCTGCTAATCGTCCACTGTGGACAATCATTAATGAACTTCTTGACCTCAAATCAGGTAGGACTACCC GCTGAACTTAAGCATATCATAG
Astraeus asiaticus	TTCTAGCATTTCGGAGTGCTGTCGCTGGCCTTTCAGGGCATGTGCACGTCTTCGGAGTGTCTGCCTTCGGACCTCCGAAC CCTCTCCTATACCTCCCAAACACACCTGTGTGCACCTGTTGTAGGCCTTGTCTATTAGGCAGACCTATGTATTACTTTCAT AAACATCGCATGTATAAAAGAATGTTTGAACACGATATATATGAATAAATATAACTTTCAGCAATGGATCTCTTGGCTC TCGCATCGATGAAGAACGCAGCGAATTGCGATAAGTAATGTGAATTGCAGATTTTCCGTGAATCATCGAATCTTTGAAC GCACCTTGCGCTCCTTGGTATTCCGAGGAGCATGCCTGTTTGAGTGTCATCGAAATCTCAAAATCCTAGCTTTGCCTTGTC GGAGCTTGGTTTTTGGACTTTGGGAGTTTGCGGGCGACCCCTTTGCTTTGGGATGTCGGCTCTCCTTAAATGTATTAGCGG

	TGGGTGCAAGTCCTTTGCATGGCACGGCCTGTTCGACGTCGTAGTGATCGTCGCGGGCTGGAAGTGCTTGGATTGACGTG<br>TCTCATGCTTCCAACTTTTACATGCGCCAAGTTTAGTCTAGGCTACTCTAGCGTGTGTCCTTTTCTCTAAGGCTTGACCTC<br>AAATCAGGTAGGACTACCCGCTGAACTTAAGCATATC
Calvatia gigantea	GGGTTGTAGCTGGCTCTTCGGGGCATGTGCACGCCTGTCTTGACTCTATTCATCCACCTGTGCACCTTTTGTAGTCTTGGG<br>GGTTGAGAGCAGTCAACTATCGGATGGCTATGTGGCCTTTCCGGATGTGAGGATTGCTGAGTGCGAAAGCATACAGCTC<br>TTCTCAAAGCGACTTGTAAACCTCTCCTTCGAGCACTATGTTTTCATATACCACATAGTATGTTGTAGAATGTGATCAATG<br>GGCCTATGTGCCTATAATAATCTTATACAACTTTCAGCAACGGATCTCTTGGCTCTCGCATCGATGAAGAACGCAGCGA<br>AATGCGATAAGTAATGTGAATTGCAGAATTCAGTGAATCATCGAATCTTTGAACGCACCTTGCGCTCCTTGGTATTCCGA<br>GGAGCATGCCTGTTTGAGTGTCATTAAATTCTCAACCCCTCCAGCTTTTGTGAGTTGCGATGGGGCTTGGAAGGTGGGAG<br>TTTGCGGGCCTCTGTAATAGAGGTCGGCTCTCCTGAAATGCATTAGCGGAACCGTTTGCAGTCCCGTCACTAGTGTGATA<br>ATTATCTACACTGTGATGATTGCTCTCCGGCTTGTTCAGCTACTAATCGTCCTTTACCGGACAATGTATAATGAACATCTT<br>GACCTCAAATCAGGTAGGACTACCCGCTGAACTTAAGCATATCA
Daedaleopsis tricolor	TGTAGCTGGCCTTCCGAGGCATGTGCACGCCCTGCTCATTCCACTCTACACCTGTGCACTTACTGTGGGTCTCAGGCGAG<br>CGTCGGTCGCTTCGCGGCGTCGTCGTTCAACTGGGCTCACGTTTTACTACAAACTATTAAAGTATCAGAATGTCTACTGC<br>GAATTAACGCATTTAAATACAACTTTCAGCAACGGATCTCTTGGCTCTCGCATCGATGAAGAACGCAGCGAAATGCGAT<br>AAGTAATGTGAATTGCAGAATTCAGTGAATCATCGAATCTTTGAACGCACCTTGCGCTCCTTGGTATTCCGAGGAGCAT<br>GCCTGTTTGAGTGTCATGAAATTCTCAACCTAACGAGTCTTTGCGGGCTCGGTAGGCTTGGACTTGGAGGTTCTTGTCGG<br>CCTAACGGTCGGCTCCTCTTAAATGCATTAGCTCGGTTCCTTGCGGATCGGCTCACGGTGTGATAATTGTCTACGCCGCG<br>ACCGTTGAAGCGTTTTGGCCGGCTTCTAATCGTCTCGTTGGAGACACTACTCTTTATGACCTTCTGACCTCAAATCAGGTA<br>GGACTACCCGCTGAACTTAAGCATATC

Fomes fomentarius	TTGTAGCTGGCCTTCCGAGGCATGTGCACGCCCTGCTCATCCACTCTACACCTGTGCACTTACTGTGGGATTTCAGGTGC<br>GTCGCTTTGCGGCGGCGTCACTCGGCCCACGTTTTCTTTACAAACTATTGAAGTAACAGAATGTTTATTGATGTAACGCA<br>TCTATAATACAACTTTCAGCAACGGATCTCTTGGCTCTCGCATCGATGAAGAACGCAGCGAAATGCGATAAGTAATGTG<br>AATTGCAGAATTCAGTGAATCATCGAATCTTTGAACGCACCTTGCGCTCCTTGGTATTCCGAGGAGCATGCCTGTTTGAG<br>TGTCATGAAATTCTCAACCTATAAACTTTTGCGGGTTTGTAGGGTTGGCTATTGGAGGCTTTTGCTGGCCCTCGTTTGAGT<br>CAGCTCCTCTCAAATGCATTAGCTTGGTTCCTTGCGGATCGGCTGTCGGTGTGATAATGTCTACGCCGCGACCGTGAAGC<br>GTTTGGAGAGCTTCTAATGGTCTCGTCAGAGACAGCTTTTATGAACTCTGACCTCAAATCAGGTAGGACTACCCGCTGA<br>ACTTAAGCATATCAA
Russula nigricans	GGCCGGCGGAAGGGCTGTCGCTGACCTTTTTCAAGGTCGTGCACGCCCGAGCGCTCTCGCCTACAATCCACCTCACCAC<br>TTGTGCATCACCGCGCGGGGCCTCCCTCTTGAGCTCGCTTCGAGAGAGGCTCGCGTTCTTCACATCACACACACACACCT<br>TTTTAGTTTTTGAATGTCATTCATTTGCGATAACGCACCATCAATACAACTTTCAACAACGGATCTCTTGGCTCTCGCATC<br>GATGAAGAACGCCAGCAAATGCGATACGTAATGTGAATTGCAGAATTCAGTGAATCATCGAATCTTTGAACGCACCTTG<br>CGCCCCTTGGCATTCCGAGGGGCACACCCGTTTGAGTGTCGTGAAATTCTCAAACCTTCTTGGTTTCTTGACCAAGATGG<br>CTTTGGACTTTGGAGGCGTTGTGCTGGCTTTGCAAAAAGTCGGCTCCTCTTAAATGCATTAGTGGGGTCCCATTTGCCAA<br>TCCCCAGGCGTGATAAGATGTTTCTACGTCTTGGGATTTGCTCTGTTCACTGGGAACCTGCTGCCAACCGTCTCGTGAGA<br>GACATCGTTCGAACTTGCTCGGCACACGAATCTTGACCTCAAATCGGGTGAGACTACCTGCTGAACTTAAGCATATCAT
Scleroderma sinnamariense	AGCCTGTCGCTCCTCGGACCTCTCCGAAGCTTCAACCTTCTTCACACCCGTGTGCACCCGCTGTAGGTCCTTCGGGATCC<br>TACGTCTTCGCTCTCGAACTCGCATGTCTACAGAATGTCCATGTCGTGTCGACCGGCGTCCGCGTCGGCGACCGCAAACC<br>TTTGTACAACTTTCAGCAATGGATCTCTTGGCTCTCGCATCGATGAAGGACGCAGCGAATCGCGATAAGTAATGTGAAT<br>TGCAGATTTTCCGTGAATCATCGAATCTTTGAACGCACCTTGCGCTCCTCGGTATTCCGAGGAGCATGCCTGTTTGAGTG<br>TCATCGAAATCTCGAACCGAAGCCTTCGATCCGTTCGAAGCTCGGTCTCGGAACGTGGGGGTCTGCGGGCGGCGACGTC<br>GGCTCCCCTCAAACGCATCAGCTGTGGGCACGAGCCCTGCGTGGCACGGCCTCCCCGACGTCGTAACGATCGTCGTGGG

CTGGAAGCGCAGTGATCGGCGATCCCATGCTTTCCAACCTTTTTCTCGAAGCTTGACCTCAAATCAGGCAGGACTACCC
GCCGAACTTAAGCATATCAATAAGCGGAGGAGAAGAAACTAACGAGGATTCCCCTAGTAACTGCGAGTGAAGCGGGA
AGAGCTCAAATTTCGAATCTGGCGGCCGTGGGTCGTCCGAGTTGTAATCTAGAGAAGCGTCTTCC

Supplementary Figures

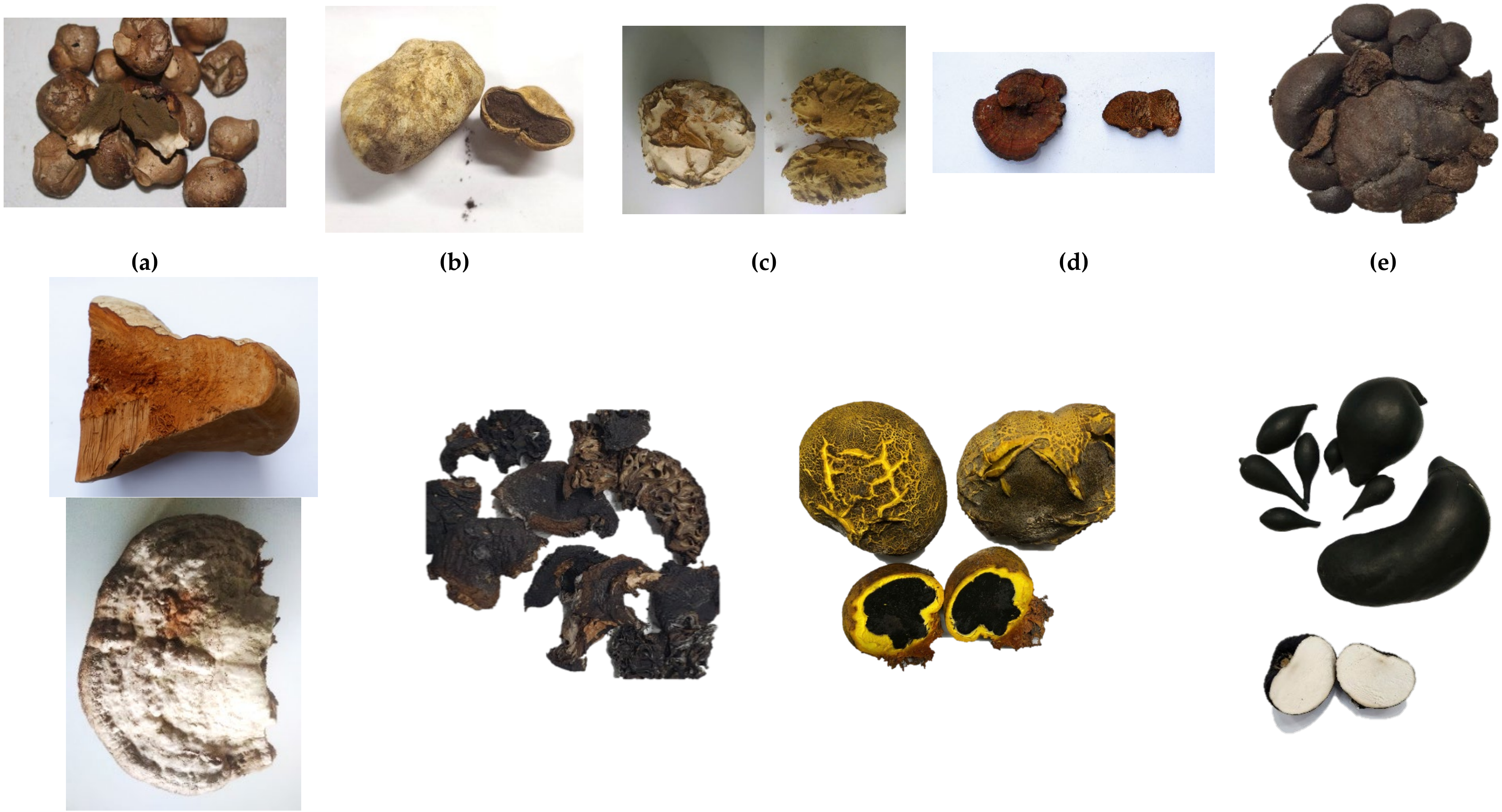

(a) (b) (c) (d) (e)

(f) (g) (h) (i)

Supplementary Figure S1. Fruiting bodies of different melanin-producing fungi. (a). *Apioperdon pyriforme*; (b). *Astraeus asiaticus*; (c). *Calvatia gigante*a; (d). *Daedaleopsis tricolor*; (e). *Daldania concentrica*; (f). *Fomes fomentarius*; (g). *Russula nigricans*; (h). *Scleroderma sinnamariense*; (i). *Xylaria nigripes*.

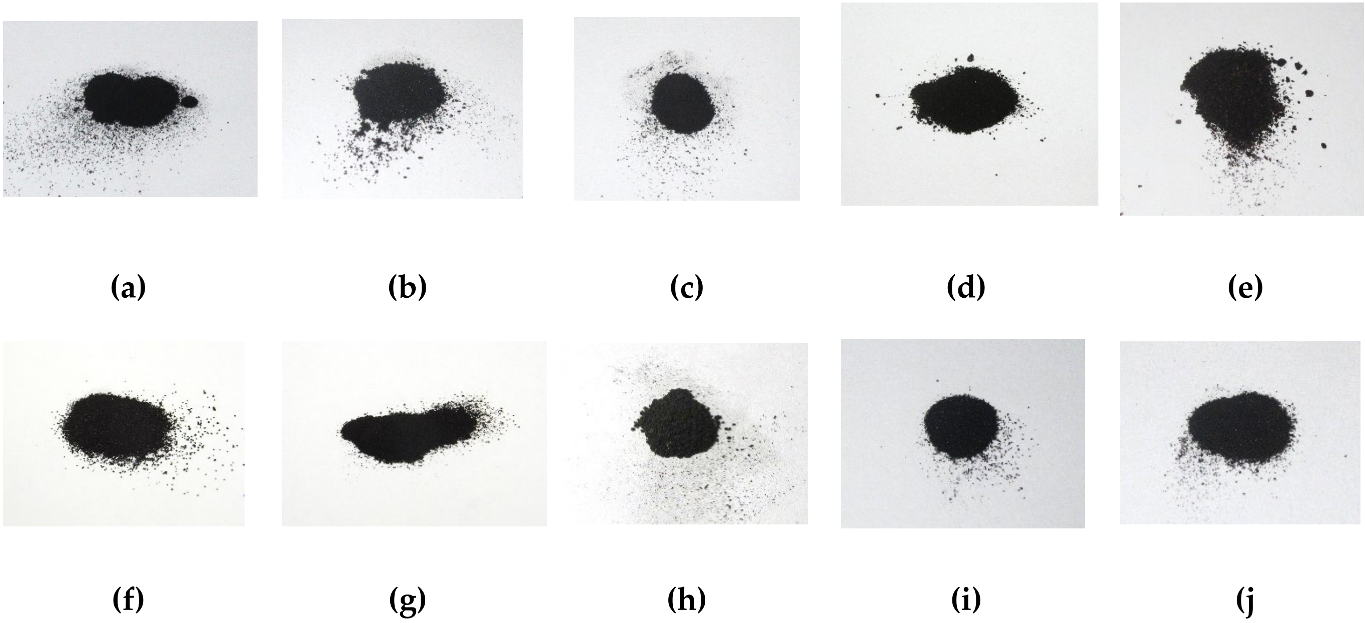

Supplementary Figure S2. Melanin pigments from the different fungal species. (a). *Apioperdon pyriforme*; (b). *Astraeus asiaticus*; (c). *Calvatia gigante*a; (d). *Daedaleopsis tricolor*; (e). *Daldania concentrica*; (f). *Fomes fomentarius*; (g). *Russula nigricans*; (h). *Scleroderma sinnamariense*; (i). *Xylaria nigripes*.

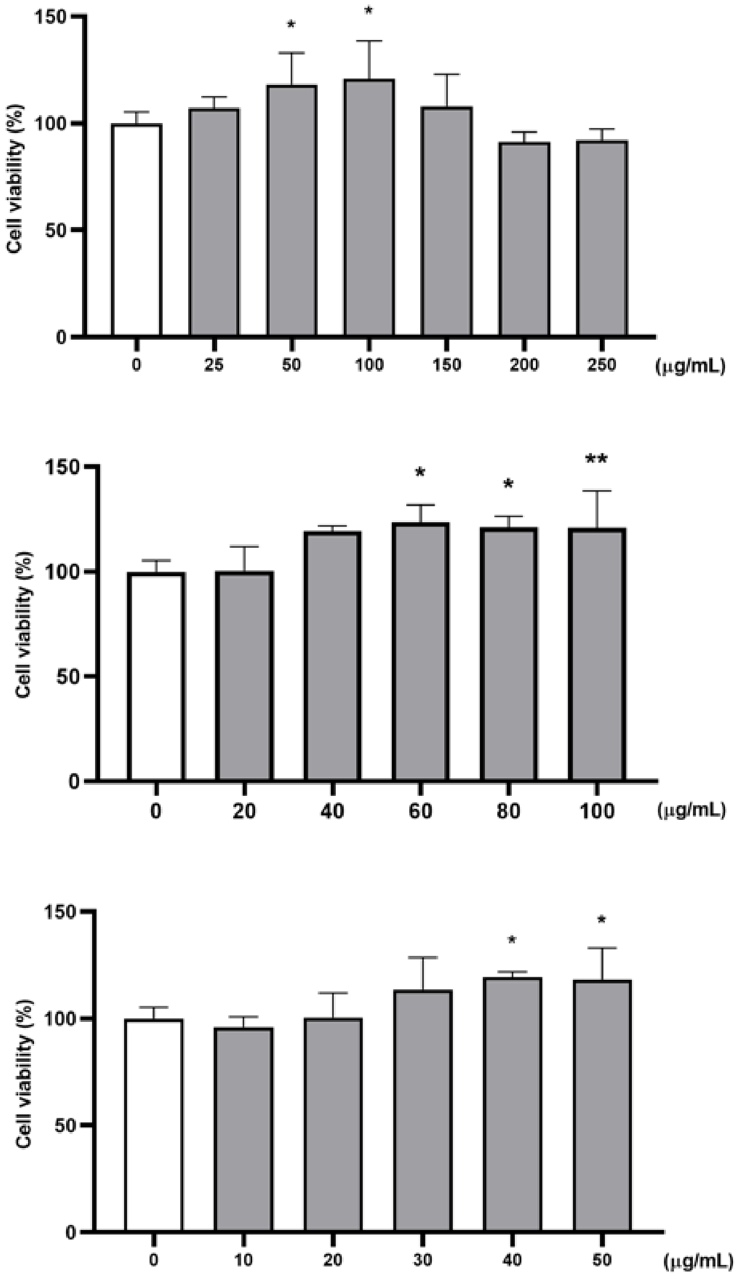

Supplementary Figure S3. Effects of different concentrations of arginine on SH-SY5Y cell viability. Different concentrations of arginine were selected based on the melanin:arginine (w/w) ratios used for the arginine modification of melanin and the tested concentrations of melanin and modified melanin on SH-SY5Y cells.

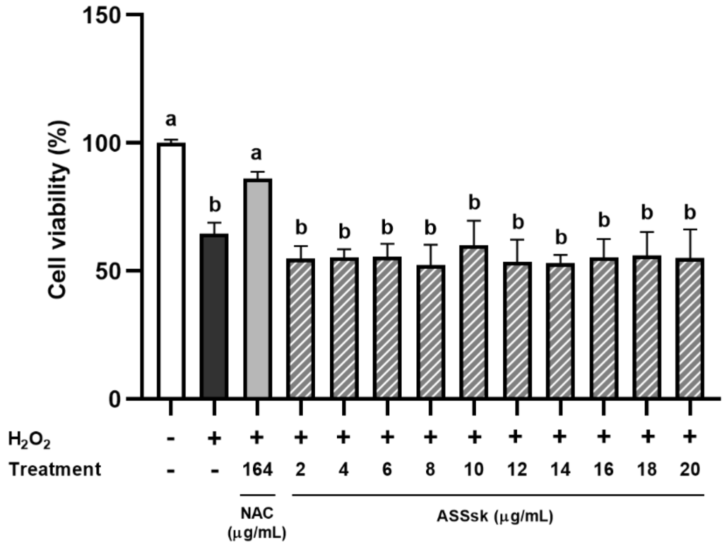

Supplementary Figure S4. Protective effects of different concentrations of arginine-modified melanin from skin of *S. sinnamariense* (**ASSsk**) against 0.6 mM H_2O_2

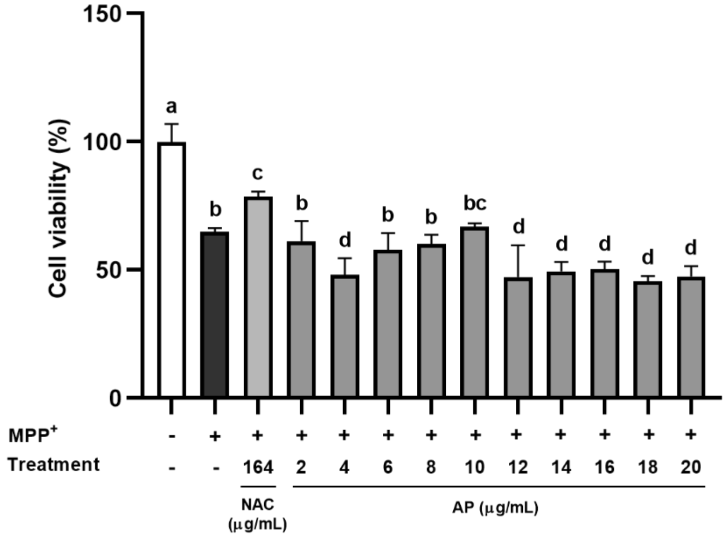

Supplementary Figure S5. Protective effects of different concentrations of melanin from *A. pyriforme* (**AP**) against 1 mM MPP^+

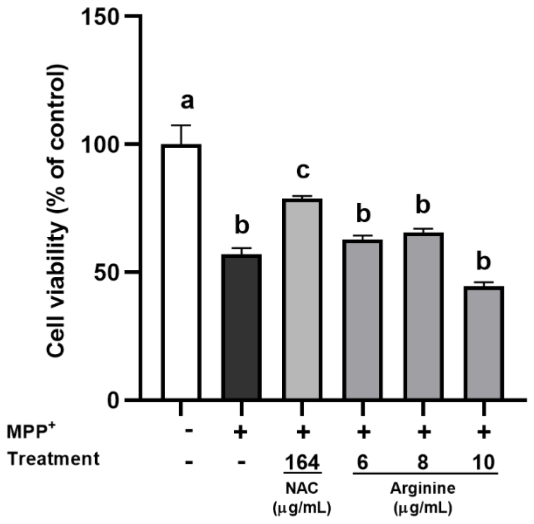

Supplementary Figure S6. Effects of arginine on cell viability of MPP^+ induced SH-SY5Y cells.
